# ptFVa (*Pseudonaja Textilis* Venom-Derived Factor Va) Retains Structural Integrity Following Proteolysis by Activated Protein C

**DOI:** 10.1161/ATVBAHA.121.316038

**Published:** 2021-06-24

**Authors:** Mark Schreuder, Xiaosong Liu, Ka Lei Cheung, Pieter H. Reitsma, Gerry A.F. Nicolaes, Mettine H.A. Bos

**Affiliations:** 1 Division of Thrombosis and Hemostasis, Einthoven Laboratory for Vascular and Regenerative Medicine, Leiden University Medical Center, the Netherlands (M.S., K.L.C., P.H.R., M.H.A.B.).; 2 Maastricht University, Department of Biochemistry, the Netherlands (X.L.).; 3 VarmX B.V, Leiden, the Netherlands (P.H.R.).

**Keywords:** activated protein C resistance, blood coagulation, factor V, snake venoms

## Abstract

**Objective:**

The Australian snake venom ptFV (*Pseudonaja textilis* venom-derived factor V) variant retains cofactor function despite APC (activated protein C)-dependent proteolysis. Here, we aimed to unravel the mechanistic principles by determining the role of the absent Arg306 cleavage site that is required for the inactivation of FVa (mammalian factor Va).

**Approach and Results:**

Our findings show that in contrast to human FVa, APC-catalyzed proteolysis of ptFVa at Arg306 and Lys507 does not abrogate ptFVa cofactor function. Remarkably, the structural integrity of APC-proteolyzed ptFVa is maintained indicating that stable noncovalent interactions prevent A2-domain dissociation. Using Molecular Dynamics simulations, we uncovered key regions located in the A1 and A2 domain that may be at the basis of this remarkable characteristic.

**Conclusions:**

Taken together, we report a completely novel role for uniquely adapted regions in ptFVa that prevent A2 domain dissociation. As such, these results challenge our current understanding by which strict regulatory mechanisms control FVa activity.

## Abstract 2

Supplemental Digital Content is available in the text.

**Highlights**

- Venom-derived factor Va from the Australian snake *Pseudonaja textilis* retains cofactor function despite proteolysis by factor Va’s natural inhibitor.
- Whereas the inactivation of human factor Va proceeds via A2 domain dissociation following limited proteolysis, A2 domain dissociation is prevented by strong noncovalent interactions in venom-derived factor Va, allowing the persistent conversion of prothrombin into thrombin.
- Molecular dynamics simulations revealed that venom-derived factor Va comprises a rigid loop in the A2 domain that forms productive interactions with the A1 domain. These interactions increase the binding affinity between the A1 and A2 domains, whereas the weak interactions in human factor Va result in A2 domain dissociation upon proteolysis.

Blood coagulation is a tightly regulated process composed of the sequential and concerted action of several serine proteases and their respective cofactors, together responsible for minimizing blood loss following vascular injury. Central to this process is the activation of FV (coagulation factor V) into the active FVa (cofactor Va).^1–3^ Once FV is partially or fully activated, it functions as cofactor for the serine protease FXa (factor Xa) by formation of the prothrombinase complex on the surface of an anionic membrane in the presence of calcium ions.^4,5^ This complex rapidly converts prothrombin to thrombin, a key regulatory enzyme in coagulation. The coagulation system can also be exploited to gain selective advantages. The procoagulant venom of several Australian Elapid snakes contains a powerful enzyme complex that specifically activates prothrombin to disrupt the prey’s intrinsic hemostatic balance.^6–9^ This prothrombin-activating complex consists of FX- and FV-like proteins^10–13^ comprising certain remarkable gain-of-function adaptations that enable this prothrombinase-like complex to initiate coagulation in an uncontrolled manner.^14–17^ Rao et al^11^ and our group^14,18^ have demonstrated that common brown snake venom ptFV (*Pseudonaja textilis* venom-derived factor V), while undergoing selective proteolysis, is functionally resistant to FVa’s natural inhibitor APC (activated protein C).

**See cover image**

To downregulate the procoagulant response, coagulant FVa is inactivated by APC via proteolysis of the A2 domain residues Arg306, Arg506, and Arg679.^19–23^ Cleavage at Arg306 and Arg506 results in A2 domain dissociation and subsequent loss of FXa affinity.^24^ While ptFVa is cleaved at a position that is homologous to human Arg506 (Lys507),^14^ an Arg306-like cleavage site is absent.^11,14^ Furthermore, venom FV comprises a unique disulfide bond that covalently links the A2 and A3 domains.^14,16^ Although we have previously reported that this disulfide bond is not responsible for the functional APC resistance,^18^ whether it might stabilize the A2 domain binding affinity upon introduction of the Arg306 cleavage site remains to be determined.

In the present study, we generated several chimeric ptFV variants with the goal of uncovering the mechanistic principles of its exceptional functional resistance to APC. Our results demonstrate that ptFV has uniquely adapted several structural elements to prevent A2 domain dissociation and ptFVa inactivation. As such, these findings highlight the remarkable evolutionary adaptations that have made ptFVa into an extremely stable procoagulant venom and provide new insights in the structural requirements of APC-induced FVa inactivation.

## Materials and Methods

The data that support the findings of this study are available from the corresponding author upon reasonable request.

### Materials and Reagents

The inhibitor dansylarginine-N-(3-ethyl-1,5-pentanediyl)amide was from Haematologic Technologies (Essex Junction, VT). The peptidyl substrate H-D-Phe-Pip-Arg-pNA (S2238) was obtained from Instrumentation Laboratories (Bedford, MA). All tissue culture reagents were from Life Technologies (Carlsbad, CA). Small unilamellar phospholipid vesicles (PCPS) composed of 75% (wt/wt) hen egg L-phosphatidylcholine and 25% (wt/wt) porcine brain L-phosphatidylserine (Avanti Polar Lipids, AL) were prepared and characterized as described previously.^25^ FV-depleted human plasma and Neoplastine CI Plus 10 prothrombin time reagent were obtained from Diagnostica Stago (Paris, France). All functional assays were performed in HEPES buffered saline (20 mmol/L HEPES, 0.15 mol/L NaCl, pH 7.5) supplemented with 5 mmol/L CaCl_2_, 0.1% PEG8000, and filtered over a 0.2 µm filter (assay buffer).

### Proteins

The human plasma-derived coagulation factors FXa, prothrombin, α-thrombin, APC, protein S, and plasmin were from Haematologic Technologies (Essex Junction, VT). Restriction endonucleases XcmI and Bsu36I were obtained from New England Biolabs (Ipswich, MA). Recombinant venom-derived ptFXa (*Pseudonaja textilis* venom-derived factor Xa) was prepared, purified, and characterized as described.^18^ Recombinant constitutively active B-domainless hFV (human factor V; FV-810) and venom-derived ptFV were prepared, purified, and characterized as described previously.^14,26^ The molecular weight and the extinction coefficients (*Ε*_0.1%, 280 nm_) of the newly generated hFV-pt306, ptFV-h306, and ptFV-h306 S-S were assumed to be equal to hFV (216 000 kDa; 1.54) and ptFV (163 000 kDa; 1.25), respectively.^14,26^

### Construction of FV Variants

Constructs encoding for hFV comprising the ptFV Arg306 region (ptFV sequence Gly303-Thr309; Uniprot: Q7SZN0), designated pED-hFV-pt306, and ptFV comprising the human Arg306 region sequence (human full-length FV sequence Pro302-Leu308; Uniprot: P12259), named pED-ptFV-h306 were generated. Subsequently, ptFV’s unique disulfide bond (ptFV residues Cys642 and Cys1002^16^; Uniprot: Q7SZN0) was removed by replacing the cysteines for serine residues, thereby creating pED-ptFV-h306 S-S. ptFV-h306 and ptFV-h306 S-S were generated employing mutagenic complementary oligonucleotides as described.^14^ The variant hFV-pt306 was generated using a pUC57 plasmid encoding pED-ptFV nucleotides 2124-2144, flanked by pED-hFV nucleotides, which was generated and purchased from Genscript (Piscataway, NJ). The resulting pUC57 plasmid was digested with XcmI and Bsu36I, gel-purified, and subcloned into pED-hFV via digestion with the same enzymes.

### Expression and Purification of FV Variants

Transfection of the plasmids encoding FV variants into baby hamster kidney cells, the selection of stable clones, and the expression and purification of hFV and ptFV were performed as described previously.^14,26^ Briefly, FV proteins were purified employing ion-exchange chromatography on a Q-Sepharose FF (hFV variants) or SP-Sepharose (ptFV variants) column (GE Healthcare) using a NaCl elution gradient in HEPES buffered saline supplemented with 5 mmol/L CaCl_2_ and 1 mmol/L benzamidine, pH 7.4. Fractions containing FV activity were pooled and dialyzed against 20 mmol/L HEPES, 5 mmol/L CaCl_2_, pH 7.4 for human FV variants or 20 mmol/L MES, 5 mmol/L CaCl_2_, pH 6.0 for ptFV variants, loaded on a POROS HQ/20 (hFV variants), or HS/20 column (ptFV variants; Applied Biosystems) and eluted using a NaCl gradient. Protein purity was assessed by SDS-PAGE analysis using precast 4% to 12% gradient gels under nonreducing and reducing conditions (50 mmol/L dithiothreitol) using the MOPS buffer system (Life technologies; Carlsbad, CA) followed by staining with Coomassie Brilliant Blue R-250. For pretreatment with thrombin, FV variants were incubated for 15 minutes with α-thrombin (molar ratio: hFV 15:1; ptFV 3.75:1) in assay buffer at 37 °C, followed by the addition of a 10-fold molar excess of hirudin over thrombin.

### Factor Va-Dependent Prothrombin Activation

Steady-state initial velocities of macromolecular substrate cleavage were determined discontinuously at 25 °C in assay buffer as described.^27,28^ Briefly, unless otherwise stated, progress curves of prothrombin were obtained by incubating PCPS (50 μmol/L), dansylarginine-N-(3-ethyl-1,5-pentanediyl)amide (10 µmol/L), and prothrombin (1.4 µmol/L) with the various recombinant FV(a) species (0-40 nmol/L), and the reaction was initiated with 0.4 nmol/L of FXa or ptFXa upon which the rate of prothrombin conversion was measured.^27^

### Activated Protein C-Mediated Inactivation of FVa Variants

For APC-mediated proteolysis, FVa variants (500 nmol/L) were first incubated at 37 °C with APC (hFVa 10 nmol/L; ptFVa 750 nmol/L) in the presence of PCPS (50 µmol/L). In addition, similar reactions containing ptFVa variants (50 nmol/L) and PCPS (50 µmol/L) were treated with 75 nmol/L APC in the presence of protein S (100 nmol/L). Following APC treatment, aliquots were taken and assayed for cofactor activity. Activity assay mixtures contained prothrombin (1.4 µmol/L), PCPS (50 µmol/L), dansylarginine-N-(3-ethyl-1,5-pentanediyl)amide (10 µmol/L), APC-proteolyzed hFVa (0.2 nmol/L) or ptFVa variants (0.1 nmol/L), and FXa (2 nmol/L) or ptFXa (1 nmol/L), respectively. Prothrombin activation was determined as described.^27^ Identification of the APC cleavage sites by N-terminal sequence analysis was performed at The Protein Facility of the Iowa State University Office of Biotechnology (Ames, IA).

### Size-Exclusion Chromatography of Activated Protein C-Treated FVa Variants

FVa variants (100–500 µg) were subjected to APC treatment (hFVa 154.2 nmol/L; ptFVa 1000 nmol/L) for 15 (hFVa) or 180 minutes (ptFVa) at 37 °C in the presence of 50 µmol/L PCPS. Reactions were quenched by the addition of 50 µmol/L PPACK and 1 mmol/L benzamidine. A 10×300 mm column containing Superdex 200 Increase (GE Healthcare) was equilibrated in assay buffer supplemented with 50 µmol/L PPACK and 1 mmol/L benzamidine. The 100 to 400 µL reaction mixture was then applied to the column and eluted with equilibration buffer at a flow rate of 0.25 mL min^−1^ at room temperature. Fractions (0.25 mL) were collected and analyzed by SDS-PAGE or Western Blot using a polyclonal antibody directed against human FV (Affinity biologicals, Ancaster, Canada) and the monoclonal antibody directed against the Asn307-Arg506 fragment (AHV-5146; Haematologic Technologies, Essex Junction, VT) as described.^29^

### Molecular Structure Preparation

For molecular dynamics (MD) simulation,^30^ the 3-dimensional structures of hFVa^31^ and ptFV (PDB ID: 4BXS) were used to build in missing loops with Loop Modeling suite in YASARA.^32^ In addition, to simulate the experimentally used ptFV variant, the original region (303-GNPDTLT-309) was exchanged for the human Arg306 region (302-PKKTRNL-308), and the unique Cys642-Cys1002 disulfide bond was disrupted. To mimic the APC-cleaved protein structure, both human and *P. textilis* FV were separated into 2 protein fragments, reflecting their state after cleavage by APC at Arg306 and Arg506/Lys507. To expedite simulations, the C1 and C2 domain structures were excluded from simulation for both the human and *P. textilis* FV structures, given that these domains are structurally nonrelated with the A2 domain.

### Molecular Dynamics

Each simulation system was solvated with an explicit TIP3P water model and neutralized by addition of Cl^−^ or Na^+^ ions. The topology and coordinate files were prepared with the Amber *ff14SB*^33^ force field. During MD simulations, 2 initial energy minimization runs were performed using a steepest descent algorithm of 50 000 steps. Subsequently, each system was gradually heated from 0 to 300 K within 50 picoseconds. This step was followed by another 50 picoseconds equilibration run with a constant temperature of 300 K. After that, a 100 ns production run was carried out using the SHAKE algorithm^34^ to constrain bonds involving hydrogen atoms. The minimization, heating, equilibration, and production runs were all applied using AMBER 16.^35^

### Binding Free Energy Calculations and Per-Residue Free Energy Decomposition Analysis

From the 100 ns production run trajectory, 2000 frames were extracted between *t*=0 to 20 ns and *t*=80 to 100 ns, to calculate binding free energies. The Molecular Mechanics/Generalized Born Surface Area method^36^ was successfully employed using igb=5 GB model in AMBER, which can be briefly summarized by the following equations:


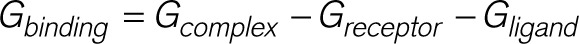
(1)


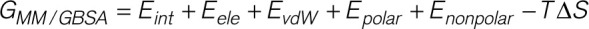
(2)

The binding free energy was calculated as the differences between free energy of the complex and the sum of the separate receptor and ligand shown in Equation 1. The relative free energy ΔGMM/GBSA was estimated by the sum of every component in Equation 2, with E_int_, E_ele_, and E_vdW_ representing internal, electrostatic and van der Waals interactions; the solvation contribution is shown with E_polar_ and E_nonpolar_. The TΔS term represents the contribution of entropy which was not taken into account when calculating relative binding free energies in our study. To determine key residues involved in binding, a per-residue energy contribution was calculated and analyzed based on the same factors used in above calculations.

### Statistical Analysis

All statistical analyses were computed using the GraphPad Prizm software. Kinetic data were analyzed by nonlinear regression using a 3-parameter logistic function or an 1-phase decay function. Statistical significance was accepted for *P* value of <0.05. All data are presented as mean±1 SD and are the result of at least 2 to 3 independent experiments, unless otherwise stated.

## Results

### Expression and Characterization of FV Variants

**Figure 1.**
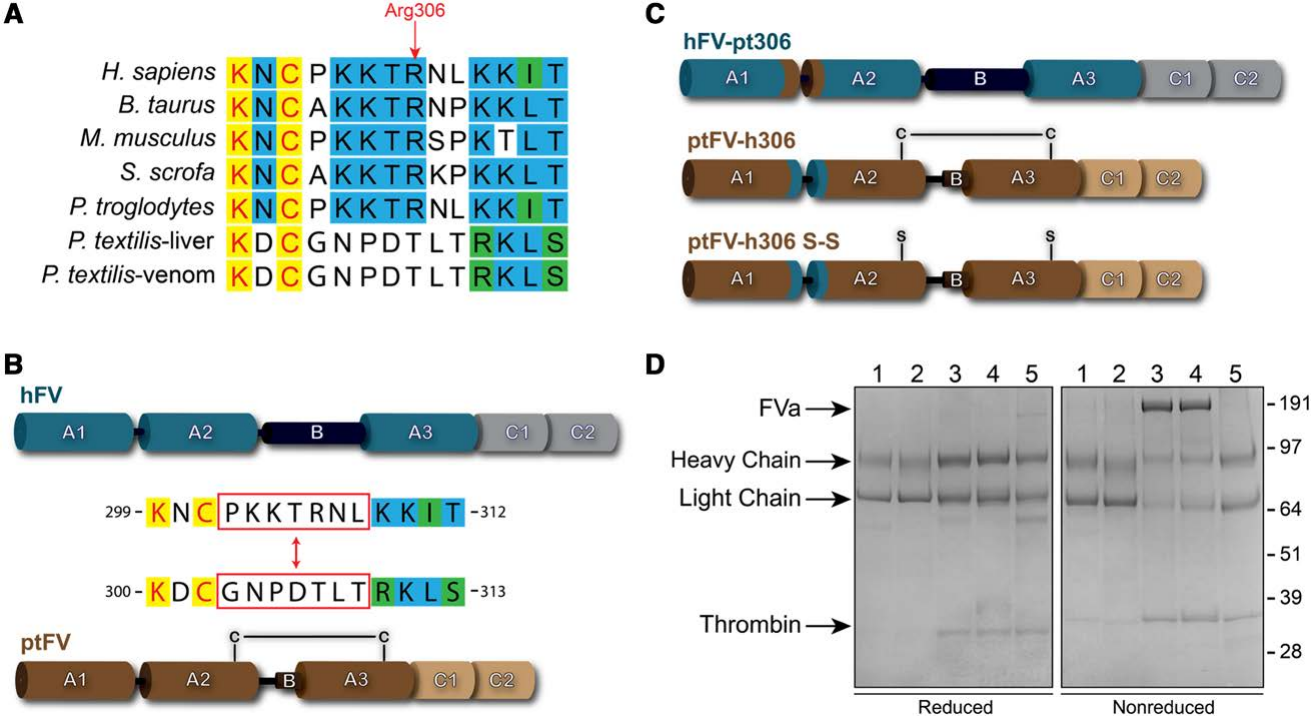
**Generation and SDS-PAGE analysis of purified factor Va variants. A**, Sequence alignment of the Arg306 region. Completely conserved residues are highlighted in yellow, partly conserved residues in blue, and residues belonging to the same classification are indicated in green. **B**, Schematic representation of B domain truncated hFV (human factor V) and venom-derived ptFV (Pseudonaja textilis factor V). The Arg306 region indicated by the red boxes was exchanged between hFV and ptFV. **C**, Schematic representation of generated FV variants. Human regions are indicated in blue/gray, ptFV regions are brown/beige. Swapping the Arg306 region generated hFV-pt306 and ptFV-h306. Additional removal of the disulfide bond by replacing the cysteines (Cys642 and Cys1002) for serines in ptFV generated ptFV-h306 S-S. **D**, SDS-PAGE of purified thrombin-activated FVa (factor Va) variants (2 µg per lane) under reducing (**left**) and nonreducing (**right**) conditions visualized by staining with Coomassie Brilliant Blue R-250. Lane 1, hFVa; lane 2, hFVa-pt306; lane 3, ptFVa; lane 4, ptFVa-h306; lane 5, ptFVa-h306 S-S. Relevant fragments and the apparent molecular weights of the standards are indicated.

Previously, we reported that ptFV retains full cofactor activity despite APC-catalyzed proteolysis.^11,14^ APC cleaves the ptFV A2 domain at Lys507 and Arg742, homologous to human Arg506 and Arg709, respectively. Intriguingly, sequence analysis revealed the lack of a potential Arg306 cleavage site and a nonconserved sequence relative to mammalian FV consisting of ptFV residues 303-GNPDTLT-309 (Figure 1A). The GNPDTLT region is well-conserved in various snakes and reptile species, suggesting that the FV Arg306 cleavage site has been introduced at a later stage in evolution relative to Arg506. As the corresponding region is strictly conserved in mammals, we exchanged the ptFV residues GNPDTLT with the corresponding human Arg306 region (302-PKKTRNL-308) of constitutively active B-domainless hFV (Figure 1B) to generate the chimeric variants hFV-pt306 and ptFV-h306 (Figure 1C). Since a unique disulfide bond in ptFV covalently links the A2 and A3 domains,^16^ this structural element could potentially prevent A2 domain dissociation and ptFVa inactivation. To exclude the contribution of this disulfide bond to ptFVa inactivation, we generated an additional variant based on ptFV-h306 in which the cysteines involved were substituted by serines, generating ptFV-h306 S-S (Figure 1C). The FV variants were stably expressed in baby hamster kidney cells and purified to homogeneity by ion-exchange chromatography. Each thrombin-activated FVa variant migrated on SDS-PAGE similar to recombinant wild-type hFVa or ptFVa (Figure 1D). In addition, SDS-PAGE analysis confirmed that the unique disulfide bond was preserved in ptFV-h306 but eliminated in ptFV-h306 S-S. Functional assessment of each FV variant revealed a similar apparent affinity for their respective FXa species (Table I in the Data Supplement), indicating that substitution of the Arg306 region does not interfere with cofactor-FXa assembly.

### ptFVa Variants Maintain Cofactor Function Upon Proteolysis by APC

**Figure 2.**
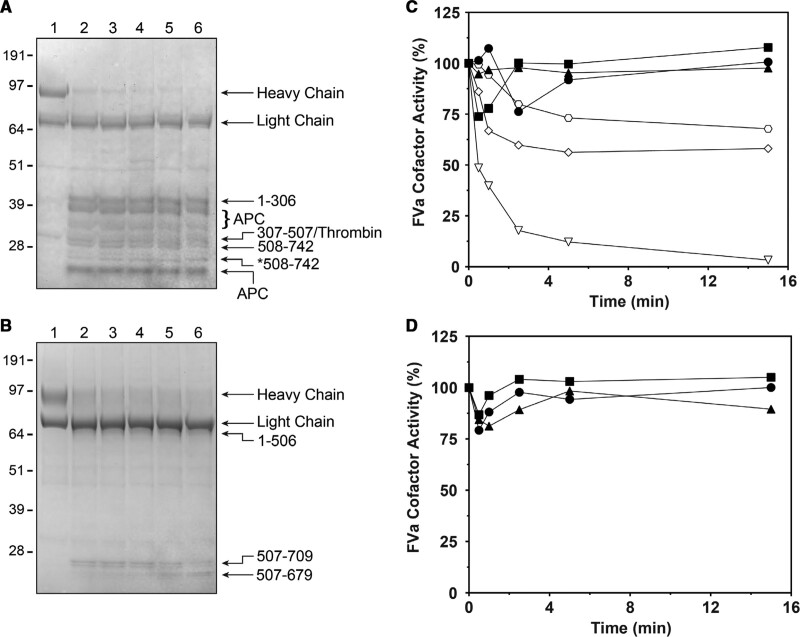
**Activated protein C-mediated proteolysis of human and Pseudonaja textilis factor Va variants. A** and **B**, SDS-PAGE of APC (activated protein C)-treated ptFVa (*Pseudonaja textilis* venom-derived factor V)-h306 (**A**) and hFVa (human factor Va)-pt306 (**B**; 3 µg per lane) under reducing conditions and visualized by staining with Coomassie Brilliant Blue R-250. Lanes 1-6 represent time samples quenched at 0, 0.5, 1, 2.5, 5, and 15 min. Relevant FVa (factor Va) fragments and the apparent molecular weights of the standards are indicated. We hypothesize that *508–742 is cleaved at a C-terminal position because its N-terminal sequence was determined to be the same as 508–742 (see Figure II in the Data Supplement). **C** and **D**, Reaction mixtures containing (**C**) 50 µmol/L PCPS and 500 nmol/L hFVa (open triangles), hFVa-pt306 (open diamonds), or hFVa-Arg306Thr (open hexagons) were incubated with 10 nmol/L APC and similar mixtures containing 500 nmol/L ptFVa (closed circles), ptFVa-h306 (closed squares), or ptFVa-h306 S-S (closed triangles) were incubated with 750 nmol/L APC. In addition, similar reactions containing (**D**) PCPS (50 µmol/L) and ptFVa variants (50 nmol/L) were treated with 75 nmol/L APC in the presence of protein S (100 nmol/L). **C** and **D**, At selected time intervals, samples were removed for cofactor activity by determining the initial velocity of prothrombin conversion. The data are representative of 3 independent experiments.

We next assessed APC-mediated proteolysis of the ptFVa variants comprising the 2 major APC cleavage sites that are essential for the inactivation of mammalian FVa.^21,24^ Thrombin-activated FVa variants were subjected to APC, and the resulting cleavage pattern was analyzed by SDS-PAGE. While in hFVa variants full proteolysis was achieved in 15 minutes by 10 nmol/L APC, high concentrations of APC (750 nmol/L) were required for significant proteolysis of the ptFVa variants. As previously described,^14^ ptFVa was cleaved at Lys507 (Figure IA in the Data Supplement), which is equivalent to human Arg506. Conversely, we observed additional protein bands consistent with the cleavage of Arg306 in ptFVa-h306 (Figure 2A). N-terminal sequencing of the cleavage products confirmed the generation of a ptFVa variant comprising the homologous Arg306 and Arg506 cleavage sites (Figure II in the Data Supplement). Removal of the unique disulfide bond in ptFVa-h306 S-S did not affect the cleavage pattern relative to ptFVa-h306 (Figure IB in the Data Supplement). Moreover, while hFVa was proteolyzed at Arg306, Arg506, and Arg679 (Figure IC in the Data Supplement), an Arg306-like cleavage was absent in hFVa-pt306 (Figure 2B), demonstrating that the GNPDTLT region in ptFV is not targeted by APC.

Subsequently, the cofactor activity of the APC-proteolyzed FVa variants was assessed. Whereas hFVa was fully inactivated within 5 minutes, functional analysis employing a purified prothrombinase assay revealed partial inactivation of hFVa-pt306 by APC, in a manner similar to the previously characterized hFVa-Arg306Thr variant^37^ (Figure 2C). Surprisingly, despite introduction of the Arg306 cleavage site and removal of the unique disulfide bond, both chimeric ptFVa variants maintained full cofactor activity during APC treatment (Figure 2C). In addition, assessment of the ptFVa cofactor activity following an initial APC treatment in the presence of protein S, a cofactor for APC,^38,39^ revealed identical results (Figure 2D). These findings indicate that the absence of a Arg306-like cleavage site and presence of ptFVa’s unique disulfide bond do not contribute to the molecular mechanism that is at the basis of ptFVa’s functional APC resistance.

### ptFV Retains Structural Integrity Following APC-Mediated Proteolysis

**Figure 3.**
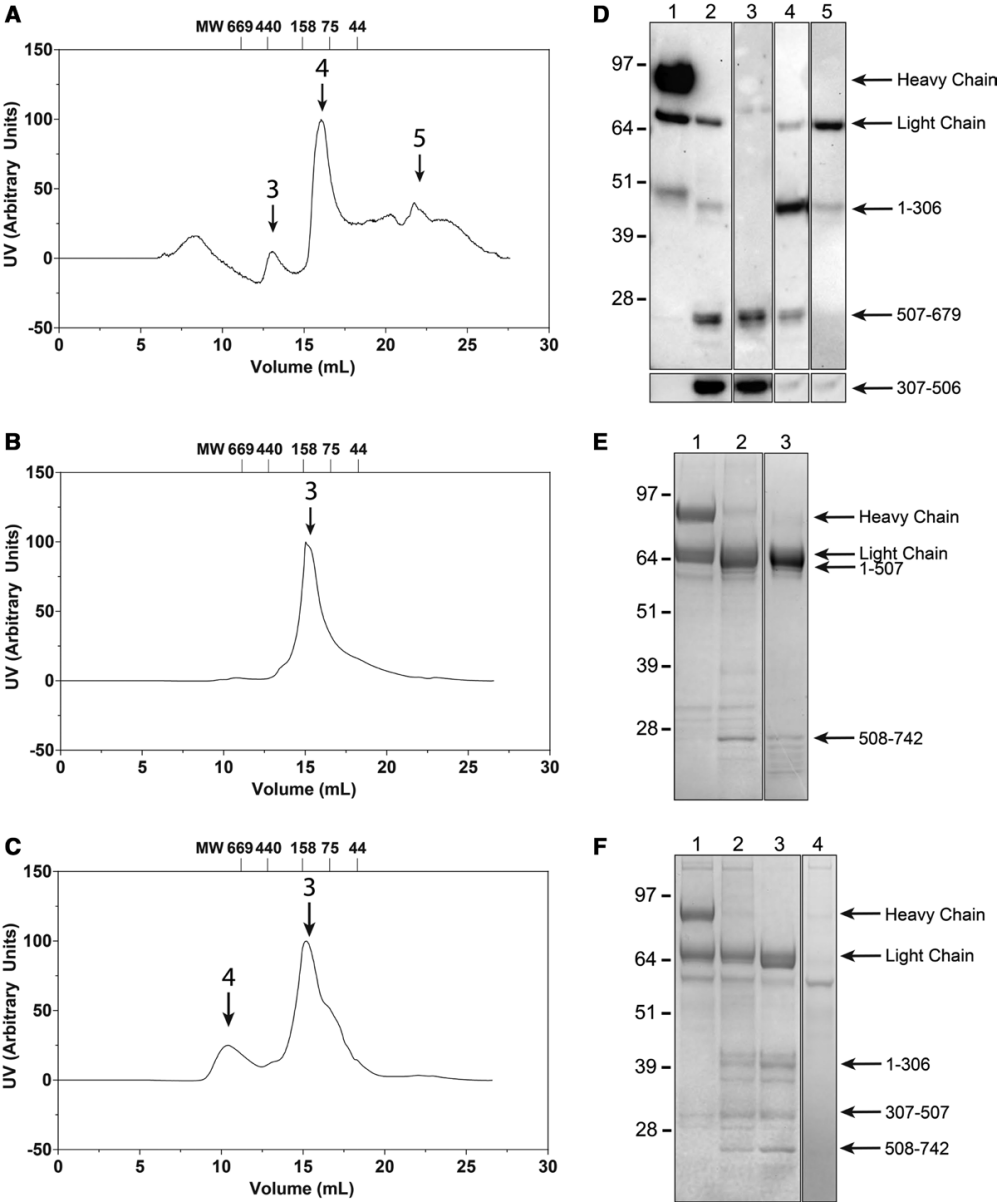
**Size-exclusion chromatography of activated protein C-treated FVa (factor Va) variants. A–C**, Size-exclusion elution profiles of APC-treated hFVa (human factor Va; **A**), ptFVa (**B**), or ptFVa-h306 S-S (**C**) performed as detailed in Materials and Methods. Arrows and numbers indicate the respective fractions shown in **D–F. D**, Western blot analysis of hFVa under reducing conditions following proteolysis by APC and size-exclusion chromatography elution. Lane 1, thrombin-activated hFVa; lane 2, APC-treated hFVa; lane 3, A2 domain fraction; lane 4, A1-A3-C1-C2, and A2 domain fraction; lane 5, A1-A3-C1-C2 domains fraction. **E** and **F**, SDS-PAGE of APC-treated ptFVa (**E**) or ptFVa-h306 S-S (**F**) following size-exclusion chromatography under reducing conditions and visualized by staining with Coomassie Brilliant Blue R-250. Lanes 1, thrombin-activated ptFVa variant; lanes 2, APC-treated ptFVa variant; lanes 3 and 4, elution fractions indicated by the black arrows and numbers in **B** and **C**. Relevant FVa fragments and the apparent molecular weights of the standards are indicated. The data are representative of 2 independent experiments. **D–F**, Composed of 2 Western Blots or SDS-PAGE gels, respectively. MW indicates molecular weight.

APC-catalyzed proteolysis at the 2 major FVa cleavage sites Arg306 and Arg506 is known to result in A2 domain dissociation.^24^ Since our ptFVa variants maintain full cofactor activity following APC treatment, we hypothesized that despite selective proteolysis, A2 domain dissociation is prevented. Initial observations employing Native-PAGE analysis suggested that APC-mediated proteolysis resulted in the fragmentation of hFV, whereas ptFV and ptFV-h306 S-S migrated as single molecules (Figure III in the Data Supplement). Intriguingly, our results show that nonproteolyzed hFV migrated as multiple species. Although the individual species could not be identified, this observation might be explained by the presence of impurities (≈30 and 51 kDa, Figure 2A). Alternatively, these results could reflect partial glycosylation at Asn2181, leading to the 71/74 kDa hFVa light chain (lane 1, Figure 2A).^40^ Nevertheless, the observation that APC-proteolyzed hFV migrates markedly different on native-PAGE relative to untreated hFV hints towards the fragmentation of the hFV protein. To explore the structural consequences of proteolysis in more detail, we determined the structural integrity of the APC-treated ptFVa variants by size-exclusion chromatography. Following APC treatment, the proteolyzed FVa variants were applied to a 10×300 mm Superdex 200 Increase column and fractions were collected and analyzed by SDS-PAGE or Western blotting. In accordance with the native-PAGE, size-exclusion profiles suggested the fragmentation of APC-treated hFVa (Figure 3A), whereas a single elution peak was observed for APC-cleaved ptFVa (Figure 3B). The elution profile of APC-treated ptFVa-h306 S-S was comparable to that of ptFVa following APC treatment (Figure 3C), although an additional elution peak was observed as a result of impurities (Figure 3F). As expected from previous observations,^24^ the 2 hFVa A2 domain fragments Asn307-Arg506 and Gly507-Arg679 migrated separately from the rest of the molecule (A1-A3-C1-C2 domains; Figure 3D). Conversely, ptFVa eluted as a single complex (Figure 3E), corroborating our functional data. Remarkably, despite introduction of the Arg306 region and deletion of the unique disulfide bond, ptFVa-h306 S-S retained structural integrity following proteolysis by APC (Figure 3F). The size-exclusion profile of ptFVa-h306 S-S displayed a shoulder in the elution peak which could indicate that the A2 domain has dissociated in a small portion of the total APC-treated FVa pool. As such, these results demonstrate that the dissociation of the ptFVa A2 domain is prevented by noncovalent interactions. Functional analysis of the chimeric FVa variants under conditions of high temperatures (Figure IVA and IVB in the Data Supplement) or high ionic strength (Figure IVC in the Data Supplement) did not reveal significant differences relative to wild-type FVa, suggestive of an enhanced A2 domain binding stability. Interestingly, proteolysis by the fibrinolytic serine protease plasmin revealed a striking decrease in ptFVa cofactor activity (Figure VA in the Data Supplement). While the loss of cofactor activity was comparable to wild-type FVa, all ptFVa variants retained up to 20% cofactor activity despite that full proteolysis was achieved within 5 minutes (Figure VB through VF in the Data Supplement). These findings provide additional support for an enhanced structural stability of essential functional regions in ptFVa.

### Stable Binding Free Energy for the A1-A2-A3 Domains of APC-Proteolyzed ptFVa-h306 S-S During MD Simulations

To gain more insight into the structural stability of human and *P. textilis* FV, 100 ns MD simulations were performed on hFVa and ptFVa-h306 S-S. The model of ptFVa-h306 S-S was generated from the X-ray structure of ptFV (PDB ID: 4BXS) in which 303-GNPDTLT-309 was substituted for the corresponding human Arg306 region and the Cys642-Cys1002 disulfide bond was disrupted. In addition, the peptide bonds that resemble Arg306 and Arg506/Lys507 cleavage were hydrolyzed in both FVa models to simulate an APC-proteolyzed state, and the C1-C2 domains were excluded from the MD simulations. Calculation of the free energies for the interaction between the A2 and A1-A3 domains revealed that these were comparable for hFVa and ptFVa-h306 S-S during the initial trajectory of the simulation (*t*=0–20 ns), with a binding free energy of −76±25 kcal/mol for hFVa and −76±14 kcal/mol for ptFVa-h306 S-S (Table 1). However, in the final part of the MD trajectory (*t*=80–100 ns), the binding free energy was remarkably increased to −39±16 kcal/mol for hFVa, whereas a substantial decrease to −90±14 kcal/mol was observed for ptFVa-h306 S-S. This suggests that over the course of the MD simulation, hFVa lost important interactions that facilitate the binding of the A2 to the A1-A3 domains. To examine these interdomain interactions in more detail, the binding free energies between the A2 and A1 or A2 and A3 domains were individually assessed. Interestingly, a loss of interaction between the A2 and A1 domains in hFVa was observed for the *t*=80−100 ns interval (4±10 kcal/mol, Table 2), while the binding free energy characterizing the A2-A3 interaction was nearly identical for both MD trajectories monitored. In contrast, the binding free energy between the A2-A1 or A2-A3 domains in ptFVa-h306 S-S remained unaffected during the simulations, indicative of a stable interaction. As such, these data confirm our in vitro studies by indicating that the structural integrity of the A1-A2-A3 domains in *P. textilis* FV is maintained following APC cleavage.

**Table 1.**
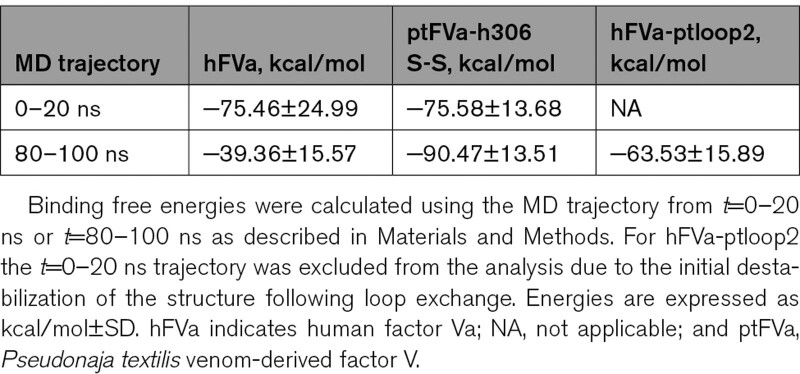
The Binding Free Energies of the A2 and A1–A3 Domain Interaction

**Table 2.**
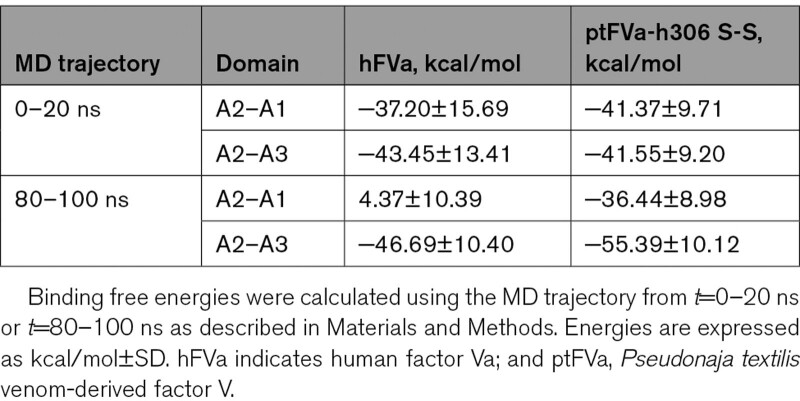
Binding Free Energies for the Binding of the A2 Domain to the A1 or A3 Domain

### Stable Hydrogen Bond Pairs Between the A2 and A1-A3 Domains Preserve A2 Domain Association in *P. textilis* FV

**Figure 4.**
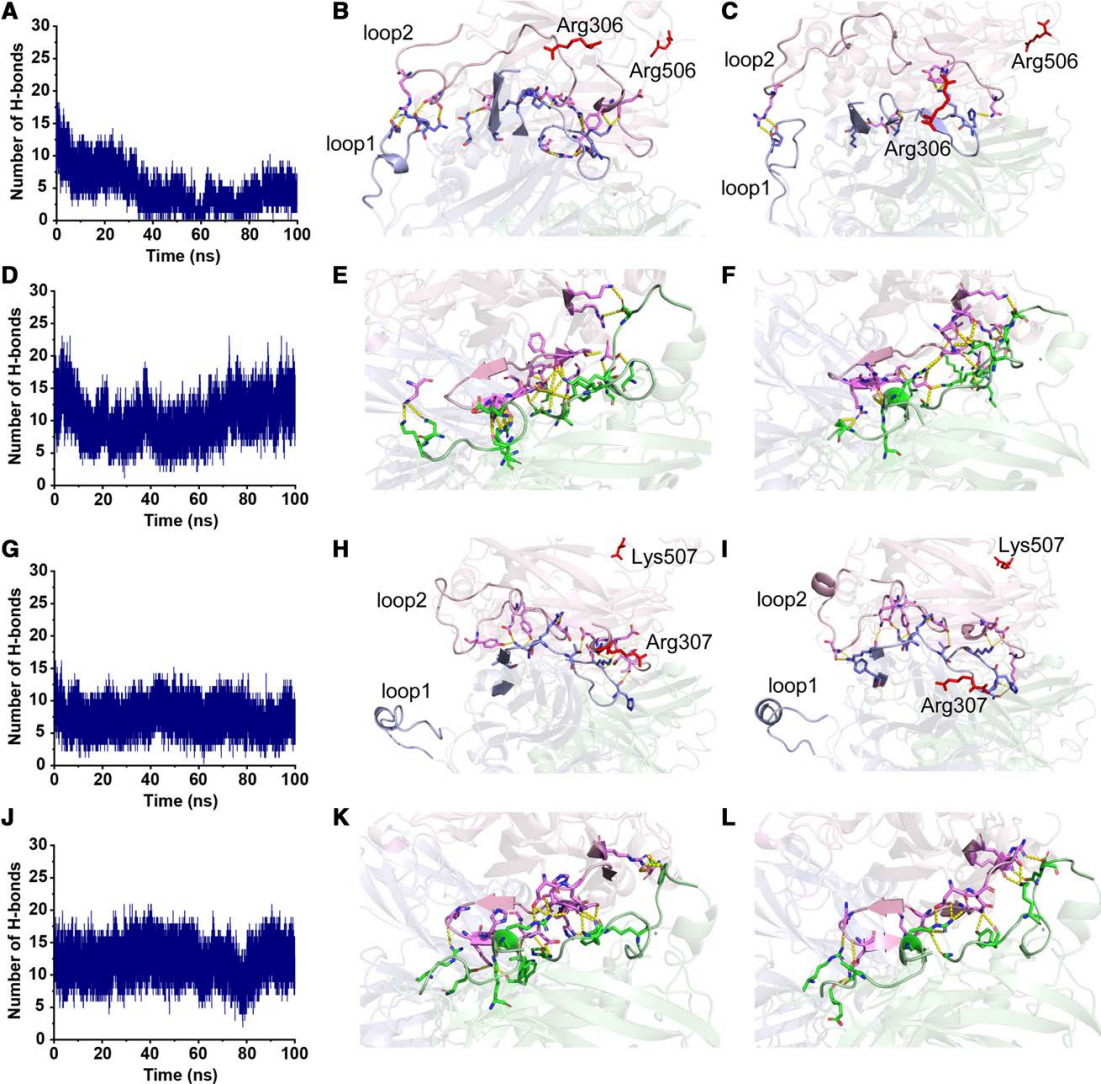
**Comparison of interdomain hydrogen bonds between surfaces of the A2 and the A1/A3 domains in human and *Pseudonaja textilis* FV (factor V).** The structural conformation and interdomain hydrogen bonds between the surfaces of the A2 and A1 (**B–C**, **H–I**) or A2 and A3 (**E–F** and **K–L**) domains are shown at the start (*t*=0 ns; **B**, **E**, **H**, and **K**) and end (*t*=100 ns; **C, F, I, L**) of the hFVa (human factor Va; **B-C, E-F**) and ptFVa-h306 S-S **(H-I, K-L**) MD simulations. The A1, A2, and A3 domains are indicated in blue, pink, and green, respectively. Residues that engage in hydrogen bond formation and the APC cleavage sites (hFVa, Arg306, Arg506; ptFVa, Arg307, Lys507) are shown in stick configuration, with the latter highlighted in red. Hydrogen bonds are indicated by the yellow dashed lines. The A1 domain loop1 and A2 domain loop2 represent the identified unique loop conformations. **A**, **D**, **G**, and **J**, Interdomain hydrogen bonds between the A2 and A1 (**A** and **G**) or A2 and A3 (**D** and **J**) domains were quantified during a 100 ns MD simulation of hFVa (**A** and **D**) and ptFVa (*Pseudonaja textilis* venom-derived factor V)-h306 S-S (**G** and **J**).

**Figure 5.**
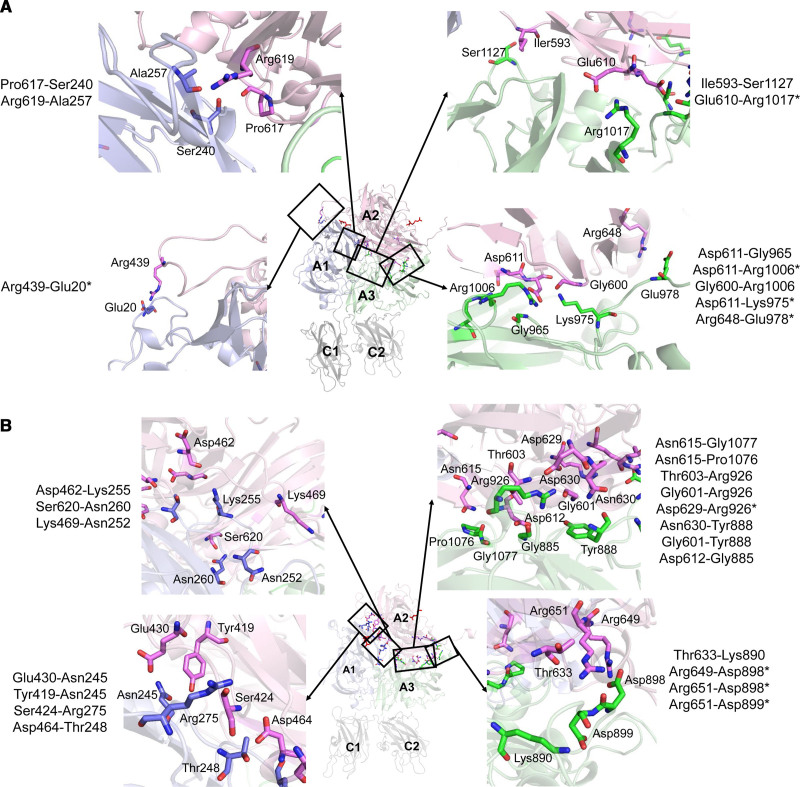
**Structural representation of residues involved in key A2-A1 or A2-A3 domain hydrogen bond interaction pairs. A** and **B**, Residues involved in key hydrogen bond interactions, as defined in Materials and Methods, between the A2 domain (pink) and the A1 (blue) or A3 domain (green) in hFVa (human factor V; **A**) or ptFV (*Pseudonaja textilis* venom-derived factor V; **B**), and the APC (activated protein C) cleavage sites (Arg306, Arg506) are shown in stick configuration, with the latter highlighted in red. The inserts display zoomed-in regions and depict the interaction pairs. The A1, A2, A3, C1, and C2 domains are indicated. *Charged interactions.

To further uncover the molecular mechanisms that are at the basis of the differential A2-A1 domain interactions observed for hFV and ptFV, the hydrogen bonds that contribute to the overall interdomain binding affinities were assessed. In accordance with the loss of A2-A1 domain interaction, the number of hydrogen bonds between the surfaces of the A2 and A1 domain in hFVa reduced over time during the MD simulations (Figure 4A). Representative structural snapshots revealed significant conformational changes in the A2 domain, suggesting that the A1 domain interacting regions are highly flexible following APC-catalyzed proteolysis (Figure 4B and 4C). In contrast, the number of hydrogen bonds formed between the A2 and A3 domains remained in a dynamic equilibrium during the simulation (Figure 4D through 4F), corroborating the stable A2-A3 binding affinity. The hydrogen bond formation between the A2-A1 and A2-A3 domains in APC-proteolyzed ptFVa-h306 S-S appeared to be balanced and most hydrogen bonds persisted during the simulation (Figure 4G through 4L). These stable interactions might be explained by the conformational constraints of the A2 domain, since only minor structural changes in the binding surfaces were observed between structures at different timepoints. Identification of residues involved in key hydrogen bond interactions during >20% of the MD simulations demonstrated ptFVa-h306 S-S to accommodate a larger fraction of these interactions relative to hFVa (Figure 5, Table II in the Data Supplement). Collectively, these findings indicate that ptFV comprises a high number of A2-A1 interactions that are preserved upon APC cleavage.

### A Unique Loop Conformation in the A2 Domain Prevents A2 Domain Dissociation in *P. textilis* FV

**Figure 6.**
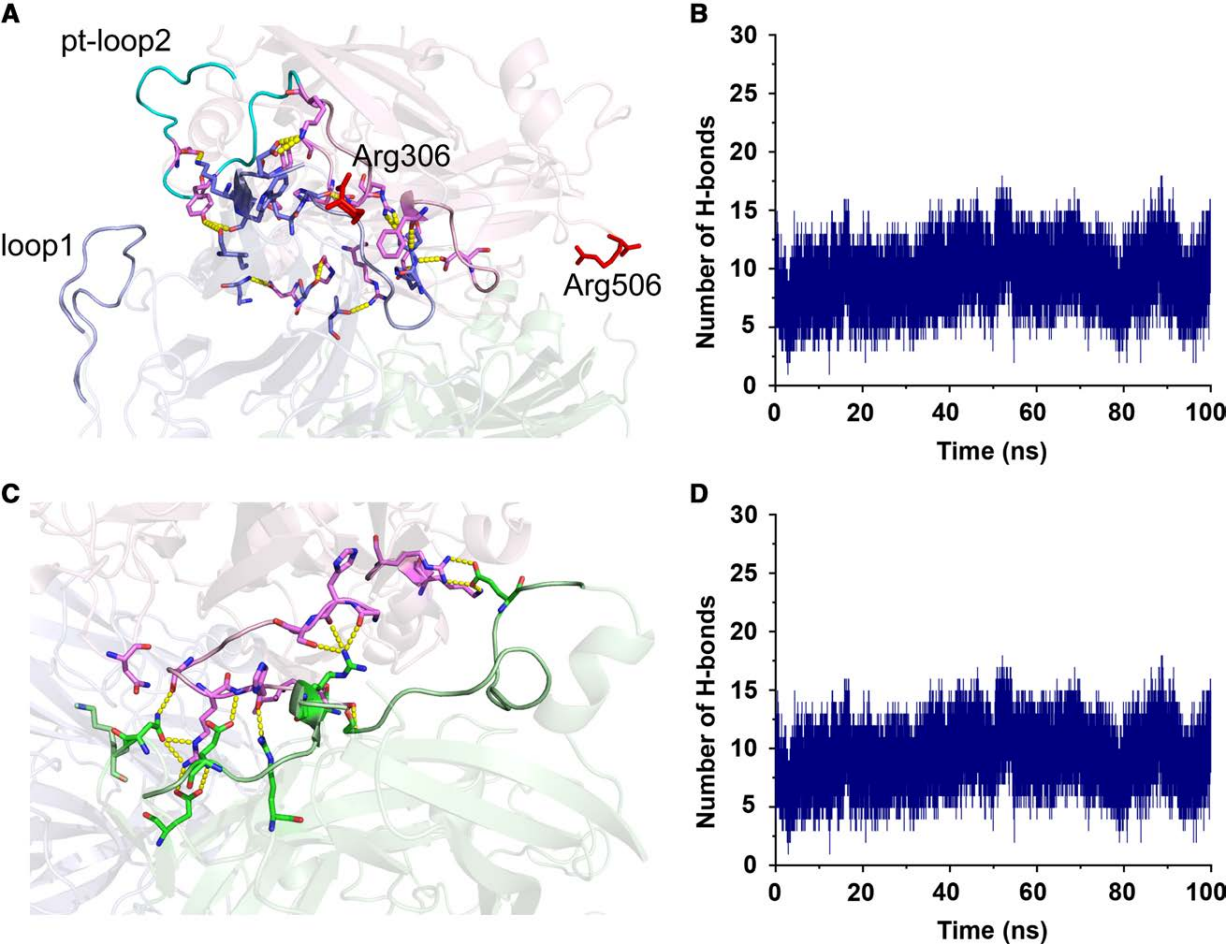
**Stable hydrogen bond formation between the A2 and A1 domains upon substitution of human loop2 for the corresponding region in *Pseudonaja textilis* FV (factor V).** In silico exchange of loop2 in hFVa (human factor Va) for the homologous loop2 in ptFV (*Pseudonaja textilis* venom-derived factor V) generated a chimeric hFVa variant. **A** and **C**, Interdomain hydrogen bonds between the surfaces of the A2 and A1 (**A**) or A2 and A3 (**C**) domains are shown in a representative snapshot of the 100 ns MD simulation of chimeric hFVa. The A1, A2, and A3 domains are indicated in blue, pink, and green, respectively. Residues that engage in hydrogen bond formation and the APC (activated protein C) cleavage sites (Arg306, Arg506) are shown in stick configuration, with the latter highlighted in red. Hydrogen bonds are indicated by the yellow dashed lines. **B** and **D**, Interdomain hydrogen bonds between the A2 and A1 (**B**) or A2 and A3 (**D**) domains were quantified during a 100 ns MD simulation of the chimeric hFVa model.

Structural comparison of the MD simulations revealed that the A2 domain of hFVa and ptFVa-h306 S-S adopt strikingly different conformations (Figure 4). In hFVa, we observed a major conformational change in a surface-exposed A2 domain loop (loop2) that is extended into the solvent and interacts with an A1 domain loop (loop1) via one stable interaction pair (Glu20-Arg439; Figure 4 and Figure VA in the Data Supplement). While human and *P. textilis* FVa share 55% sequence identity, the positioning of loop1 and loop2 is conserved (Figure VI in the Data Supplement). In addition, sequence analysis showed that loop2 is well-conserved in reptiles, whereas a significantly different sequence is conserved in mammalian FV (Figure VII in the Data Supplement). Interestingly, MD simulations in ptFVa-h306 S-S indicated that loop2 is more compact and does not interact with loop1 (Figures 4 and 5B). Supporting the structural observations, assessment of the solvent accessible surface area of individual A domains or A domain complexes over the MD trajectories further revealed that the solvent accessible surface areas of ptFVa-h306 S-S were smaller relative to hFVa (Figure VIII in the Data Supplement). This was found to be the result of a more compact A2 domain, as compared with the A1-A3 domains. To specifically determine the role of loop2 in A2 domain stability, we created an in silico chimeric hFVa structure in which the human loop2 was exchanged for the homologous ptFV loop2 region (hFVa-ptloop2), upon which a 100 ns MD simulation was performed (Figure 6A). As substitution of loop2 destabilized the structure and required several nanoseconds to adopt a stable state, we excluded the initial 20 ns from our analysis. Intriguingly, the A2 domain binding free energy in hFVa-ptloop2 was markedly decreased relative to hFVa (hFVa-ptloop2, −64 kcal/mol; hFVa, −39 kcal/mol), although it remained higher compared with ptFVa-h306 S-S (Table 1). In similar fashion, exchange of loop2 substantially decreased the average A2 domain solvent accessible surface area (15 846.2±0.05 Å^2^) as compared with hFVa (16689.3±0.04 Å^2^), but remained larger relative to ptFVa-h306 S-S (14761.8±0.04 Å^2^), illustrating the compact conformation of the ptFV loop2. Consistent with these findings, the A2-A1 domain hydrogen bonds were stabilized in chimeric hFVa-ptloop2, while the exchange of loop2 did not affect the interactions between the A2 and A3 domains (Figure 6B through 6D). Nonetheless, we cannot exclude that differences in hydrogen bond pairing between the A2 and A3 domains contribute to the decreased binding free energy for A2 domain binding in hFVa-ptloop2. Collectively, this structural bioinformatics analysis indicates that the compact and inflexible ptFV loop2 stabilizes the surface interactions between the A2 and A1 domains following APC proteolysis and could provide a mechanism that prevents A2 domain dissociation in ptFVa.

## Discussion

One of the essential characteristics underlying any hemostatic response is the timely inactivation of proteases and cofactors following proteolytic activation of their respective substrate. The specialized mechanisms of the anticoagulant system thereby prevent uncontrolled blood clotting and life-threatening thrombotic events. Our work, among others,^11,14,18^ highlights the importance of strict regulatory pathways for FV and shows that bypassing these mechanisms can be used as a potent weapon to gain evolutionary advantages.

We previously speculated that absence of the Arg306 cleavage site, required for the inactivation of mammalian FVa, is at the basis of the functional APC-resistance in ptFVa.^14,18^ To test this hypothesis, chimeric ptFV variants were generated that lack the unique A2-A3 domain disulfide bond and comprise the human Arg306 region. Our functional assessments show that despite Arg306 cleavage and disruption of the unique disulfide bond, ptFVa retains cofactor activity upon APC-catalyzed proteolysis. As such, our findings demonstrate that these elements in ptFV do not play a role in the functional resistance to APC.

Liver-derived ptFV shares 96% sequence identity to its venom paralog,^41^ and we previously revealed that liver-expressed ptFV shares the procoagulant characteristics of venom-derived ptFV, including the functional resistance to APC.^18^ These observations raise fundamental questions on the hemostatic regulatory mechanisms in *P. textilis*. It is currently unknown whether Elapidae express protein C, but if so, it would be of interest to characterize the mechanistic principles that hallmark the anticoagulant response in these snakes. It is also important to note that Arg306 is only conserved in mammals, whereas lower vertebrates do not seem to comprise an homologous Arg306 cleavage site. This indicates that lower vertebrates have a distinct mechanism to inactivate FVa. It has been suggested that reptiles have a primitive intrinsic coagulation pathway and a complete lack of factor XI and factor XII.^42–46^ The absence of these factors may favor a more procoagulant system that is required to generate sufficient amounts of thrombin. Interestingly, our findings revealed that similar to human FVa,^47,48^ plasmin was able to proteolyze and significantly reduce the cofactor activity of ptFVa, although a 20-fold higher plasmin concentration was required to obtain fully proteolyzed ptFVa. Considering that the plasma concentration of plasminogen is ~2 µmol/L,^49^ these results could suggest that plasmin plays a physiological role in the regulation of liver-expressed ptFVa. Since the generation of plasmin is taking place at a later stage of hemostasis relative to the activation of protein C, these results may support the hypothesis that liver-expressed ptFV has a more procoagulant role. In contrast, liver- and venom-derived ptFX share ≈76% sequence identity.^50^ This may indicate that venom-derived ptFX underwent evolutionary adaptations to circumvent negative regulatory mechanisms and could potentially suggest that the activity of the liver-expressed ptFVa-ptFXa complex is primarily regulated by antithrombin or tissue factor pathway inhibitor.

Using size-exclusion chromatography, we showed that despite APC-mediated proteolysis A2 domain dissociation in ptFVa is prevented. Remarkably, the A2 domain remained associated following introduction of the Arg306 cleavage site in ptFVa. Attempts to weaken the noncovalent structural integrity of the APC-treated ptFVa variants failed to significantly impact their ability to function as cofactor for ptFXa. These results demonstrate that following selective proteolysis ptFVa maintains structural integrity mediated by strong noncovalent interactions. Strikingly, these findings contradict our current understanding of FVa inactivation^51^ and imply the presence of unique structural elements that prevent A2 domain dissociation in ptFVa.

In an attempt to unravel the mechanistic principles of A2 domain association, we identified a potential crucial role for A1-A2 domain interactions. In hFVa, the binding free energy between the A1 and A2 domain increases drastically upon APC-mediated proteolysis. As a consequence, the interaction between the A1 and A2 domain is lost which seemingly triggers the dissociation of the A2 domain. The loss of A1-A2 domain binding affinity coincides with a major conformational change in the hFVa loop2. This highly flexible loop engages in weak interactions with the A1 domain loop1 and facilitates several A1-A2 domain hydrogen bonds. Upon APC-catalyzed proteolysis, the number of hydrogen bonds between the surfaces of the A2 and A1 domains reduces as a result of the conformational changes in the A2 domain. Conversely, the A1-A2 domain binding free energy remains stable following APC-catalyzed cleavage of ptFVa. In contrast to hFVa, the ptFVa loop2 does not interact with loop1 and adopts a rigid conformation that is stabilized by several key hydrogen bonds. Exchange of the ptFV loop2 to hFVa significantly improved the binding affinity between the A2 and A1-A3 domains, providing further support for the central role of loop2 in the A2 domain association. Interestingly, loop2 is located directly distal from the Arg306 cleavage site, which may suggest that the stable noncovalent interactions are able to compensate for Arg306 cleavage. Additionally, it is possible that ptFXa comprises a structural element that supports the functional APC resistance of ptFVa. Productive interactions between the proteolyzed ptFVa A2 domain and ptFXa could stabilize the conformation of ptFVa in such way that it is able to fulfill its role as cofactor. Moreover, the anionic membrane layer could be another stabilizing factor that allows for cofactor activity following APC-proteolysis. Although the ptFVa-ptFXa complex is able to convert prothrombin in the absence of membranes, it is currently unknown whether functional resistance to APC is maintained when anionic phospholipids are absent. Previous reports proposed that binding of FVa to anionic phospholipids induces conformational changes in the A2 domain that present a FXa interaction site.^52,53^ Whether such interactions are required for the procoagulant enhancements in ptFVa remains to be determined.

Overall, characterization of the mechanistic principles and identification of key regions or residues responsible for ptFV’s functional resistance to APC could provide unique opportunities for the generation of novel stable FV variants. Our findings might suggest that substitution of human loop2 for the corresponding region in ptFV could create a human FV variant with an improved intrinsic stability resulting from the increased A1-A2 domain binding affinity. Such a FV variant could have an additional advantage over previously reported Arg306Gln/Arg506Gln FV variants, since it will retain procoagulant activity independent of potential APC cleavage. It has been reported that mutation of the 3 APC cleavage sites in FVa results in FVa inactivation via APC-mediated proteolysis at alternative positions in the A2 domain.^29,54^ Moreover, FV has an important role as cofactor for APC in the inactivation of factor VIIIa,^55^ a crucial reaction to downregulate the procoagulant response.^56^ This anticoagulant role is dependent on the cleavage of Arg506 by APC^57,58^ and as such this function is absent in Arg506-mutated FV variants.

In conclusion, our findings highlight the exceptional structural features that have converted ptFV into an extremely procoagulant protein. Here, we demonstrated that ptFVa maintains functional resistance to APC despite introduction of the Arg306 cleavage site, in sharp contrast to the APC response of mammalian FVa. To do so, ptFV has uniquely adapted noncovalent interactive regions in the A1 and A2 domains that stabilize the A2 domain following proteolysis by APC. Taken together, our findings provide evidence for a unique structural element by which ptFV circumvents the regulatory mechanisms and shed new insights on the mechanistic principles of A2 domain dissociation and FVa inactivation.

## Acknowledgments

M. Schreuder, X. Liu, G.A.F. Nicolaes, and M.H.A. Bos designed the research; M. Schreuder and K.L. Cheung performed experiments. X. Liu performed in silico simulations. M. Schreuder, X. Liu, K.L. Cheung, P.H. Reitsma, G.A.F. Nicolaes, and M.H.A. Bos analyzed the data; M. Schreuder wrote the article and X. Liu, K.L. Cheung, P.H. Reitsma, G.A.F. Nicolaes, and M.H.A. Bos reviewed and revised the article.

## Sources of Funding

This work was financially supported by the Bayer Hemophilia Awards Program (Special Project Award), Landsteiner Foundation for Blood Transfusion (LSBR, grant. No. 1451), and the China Scholarship Council (to X. Liu). The funding agencies had no role in the preparation, review, or approval of the article.

## Disclosures

P.H. Reitsma owns equity in VarmX B.V. The other authors report no conflicts.

## Supplementary Material


