# Supplementary material for: ptFVa (Pseudonaja Textilis Venom-Derived Factor Va) Retains Structural Integrity Following Proteolysis by Activated Protein C
Source: Arterioscler Thromb Vasc Biol. 2021 Jun 24;41(8):2263–76. doi: 10.1161/ATVBAHA.121.316038 (PMC8288481; doi:10.1161/ATVBAHA.121.316038)
Supplement: Supplementary file 1 [file atv-41-2263-s001.pdf]

SUPPLEMENTAL MATERIAL

Pseudonaja textilis venom-derived factor Va retains structural integrity following proteolysis by activated protein C

Authors: Mark Schreuder[1†], Xiaosong Liu[2†], Ka Lei Cheung[1], Pieter H. Reitsma[1,3], Gerry A.F. Nicolaes[2], and Mettine H.A. Bos[1*]

[1] Division of Thrombosis and Hemostasis, Einthoven Laboratory for Vascular and Regenerative Medicine, Leiden University Medical Center, Leiden, The Netherlands.

[2] Maastricht University, Department of Biochemistry, Maastricht, The Netherlands.

[3] VarmX B.V, Leiden, The Netherlands.

[†] Authors contributed equally to this study.

Running title: Snake venom FV prevents A2 domain dissociation

[*] **Corresponding author:** Mettine H.A. Bos, Ph.D., Division of Thrombosis and Hemostasis, Einthoven Laboratory for Vascular and Regenerative Medicine, Leiden University Medical Center, Room C7-14, Albinusdreef 2, 2333 ZA, Leiden, The Netherlands. Email: M.H.A.Bos@lumc.nl, phone nr: +31715268133.

Supplemental Methods

Plasmin-mediated inactivation of FVa variants: Plasmin-mediated proteolysis was achieved using FVa variants (500 nM) in the presence of PCPS (50 µM) and addition of plasmin (hFVa 20 nM; ptFVa 400 nM) at 37 °C. Aliquots of the reaction mixtures were withdrawn at the indicated time intervals and analyzed by SDS-PAGE and through assessment of the residual FVa cofactor activity. Activity assay mixtures contained prothrombin (1.4 µM), PCPS (50 µM), DAPA (10 µM), plasmin-proteolyzed hFVa (0.2 nM) or ptFVa variants (0.1 nM), and FXa (2 nM) or ptFXa (1 nM), respectively. Prothrombin activation was determined as described[1].

Thermal decay of APC-treated FVa variants: Thrombin-activated ptFV variants (100 nM) were treated with APC (150 nM) in the presence of PCPS (50 µM) for 15 minutes at 37°C and were subsequently incubated in a water bath at 52°C. At indicated timepoints, an aliquot was withdrawn and diluted in assay buffer on ice. Cofactor activity of FVa aliquots were measured using assay mixtures containing prothrombin (1.4 µM), PCPS (50 µM), DAPA (10 µM), APC-proteolyzed ptFVa (0.2 nM) and ptFXa (1 nM). Prothrombin activation was determined as described[1].

Stability of APC-treated FVa variants at increased ionic strength: Thrombin-activated ptFVa-h306 S-S (50 nM) was treated with APC (75 nM) for 5 hours at 37°C in the presence of PCPS (50 µM) and increasing concentrations of NaCl (150-2000 mM) in assay buffer. Cofactor activity of FVa aliquots were measured using assay mixtures containing prothrombin (1.4 µM), PCPS (50 µM), DAPA (10 µM), APC-proteolyzed ptFVa-h306 S-S (0.1 nM) and ptFXa (1 nM). Prothrombin activation was determined as described[1].

Native PAGE: The structural integrity of FV variants was assessed by Native-PAGE using pre-cast 3-12% Bis-Tris gels (Life technologies; Carlsbad, CA, USA). APC-proteolyzed FV variants were loaded on gels using Native Sample Buffer (Life technologies; Carlsbad, CA, USA) followed by staining with Coomassie Brilliant Blue R-250.

Supplemental Figures

Supplementary Figure I

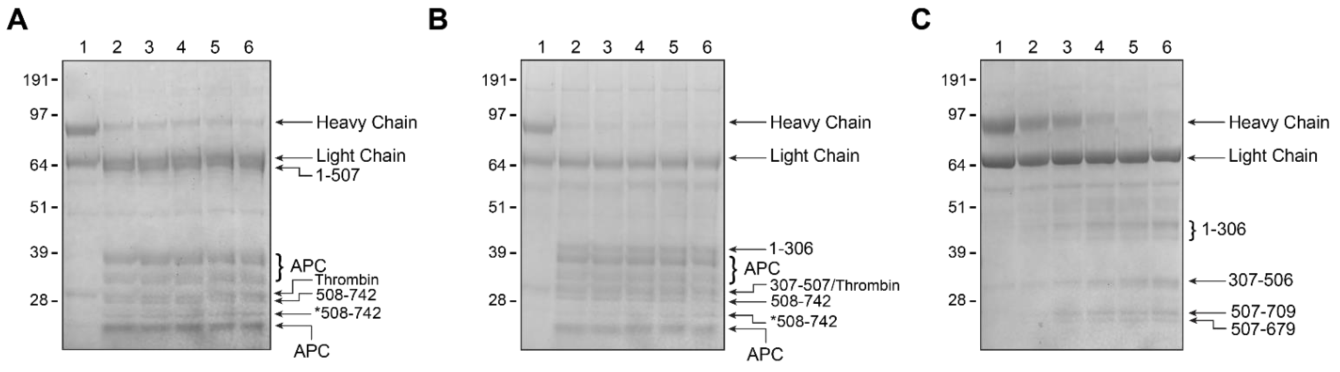

Supplementary Figure I. Activated protein C (APC) treatment of human and *P. textilis* factor Va variants. SDS-PAGE of APC-treated ptFVa **(A)**, ptFVa-h306 S-S **(B),** or hFVa **(C)** (3 µg per lane) under reducing conditions and visualized by staining with Coomassie Brilliant Blue R-250. Lanes 1-6 represent time samples quenched at 0, 0.5, 1, 2.5, 5, and 15 min. Relevant fragments including the heavy chain (A1-A2) and light chain (A3-C1-C2) and the apparent molecular weights of the standards are indicated. We hypothesize that *508-742 is cleaved at a C-terminal position because its N-terminal sequence was determined to be the same as 508-742 (see Supplementary Figure 2). The data are representative of two to three independent experiments.

Supplementary Figure II

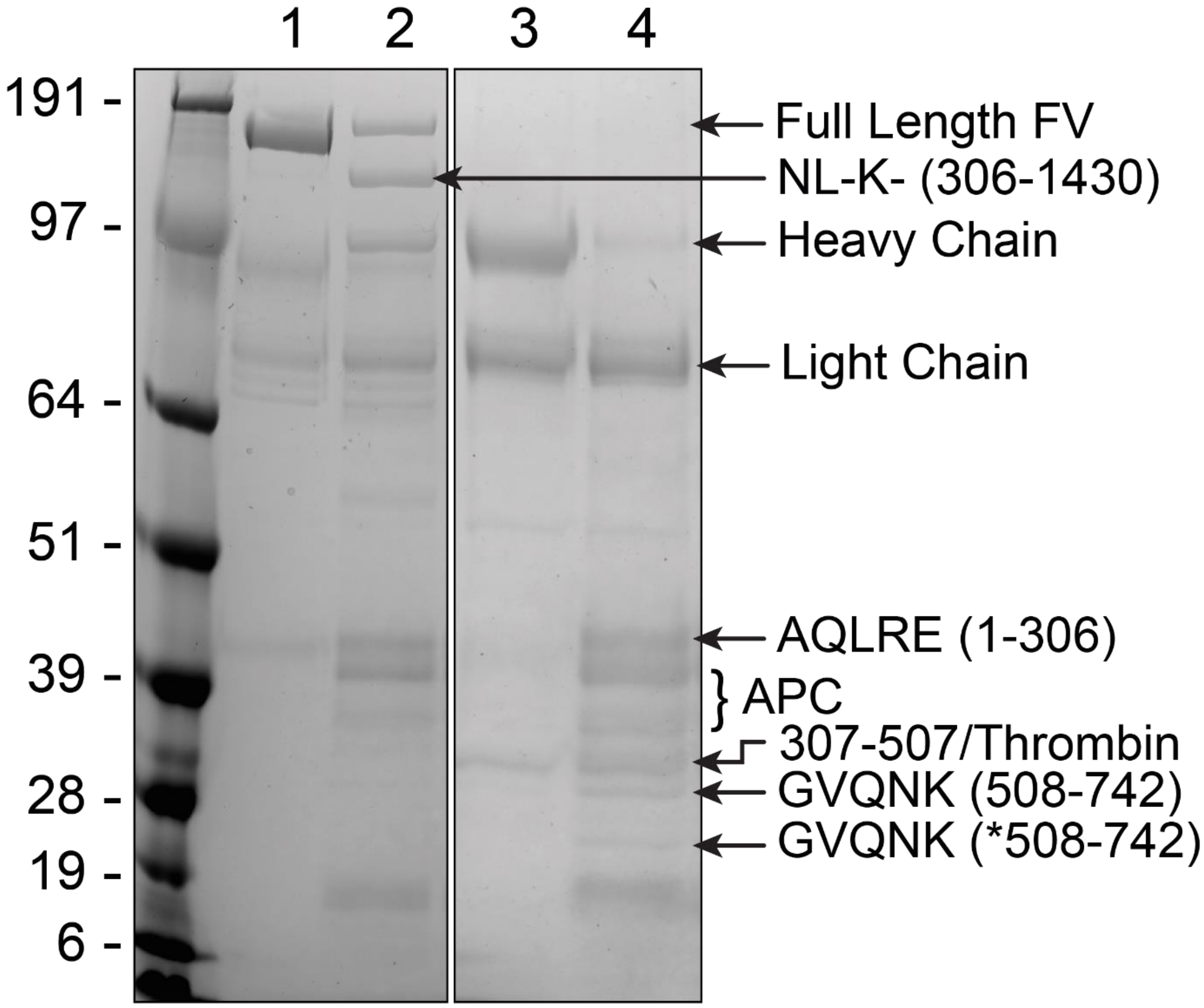

Supplementary Figure II. Characterization of APC cleavage sites in ptFV-h306. SDS-PAGE of APC-treated ptFV-h306 and ptFVa-h306 (3 µg per lane) under reducing conditions and visualized by staining with Coomassie Brilliant Blue R-250. Lane 1, ptFV-h306 at t=0; lane 2, ptFV-h306 at t=0.5min; lane 3, ptFVa-h306 at t=0; lane 4, ptFVa-h306 at t=0.5min. N-terminal sequence results of the indicated protein bands and the apparent molecular weights of the standards are shown.

Supplementary Figure III

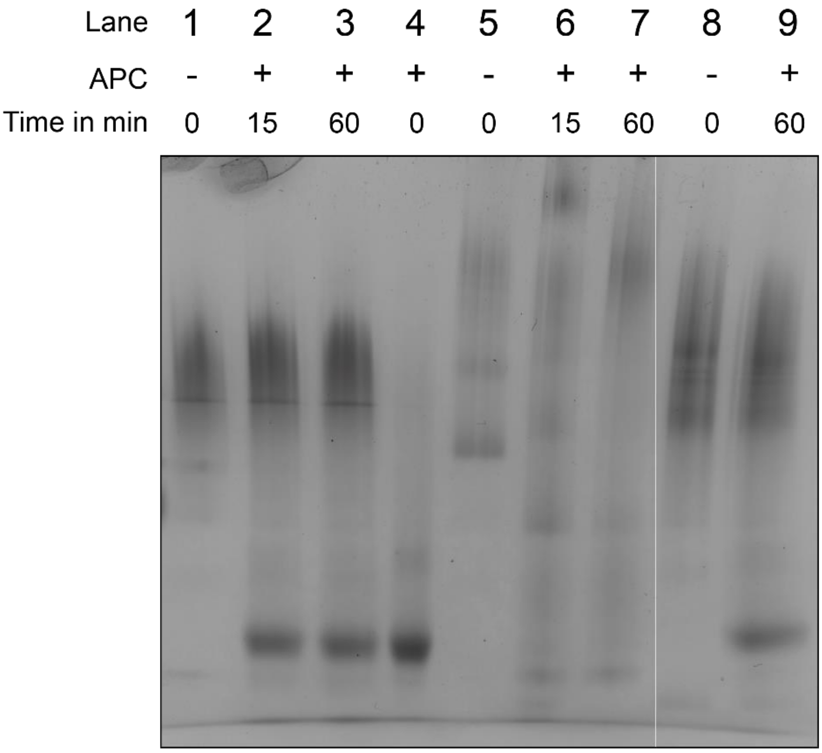

Supplementary Figure III. Native-PAGE analysis of APC-proteolyzed human and *P. textilis* factor V variants. Lane 1, ptFV at t=0 min; lane 2, APC-treated ptFV at t=15 min; lane 3, APC-treated ptFV at t=60 min; lane 4, APC; lane 5, hFV at t=0 min; lane 6, APC-treated hFV at t=15 min; lane 7, APC-treated hFV at t=60 min; lane 8, ptFV-h306 S-S at t=0 min; lane 9, APC-treated ptFV-h306 S-S at t=60 min. The data are representative of two independent experiments.

Supplementary Figure IV

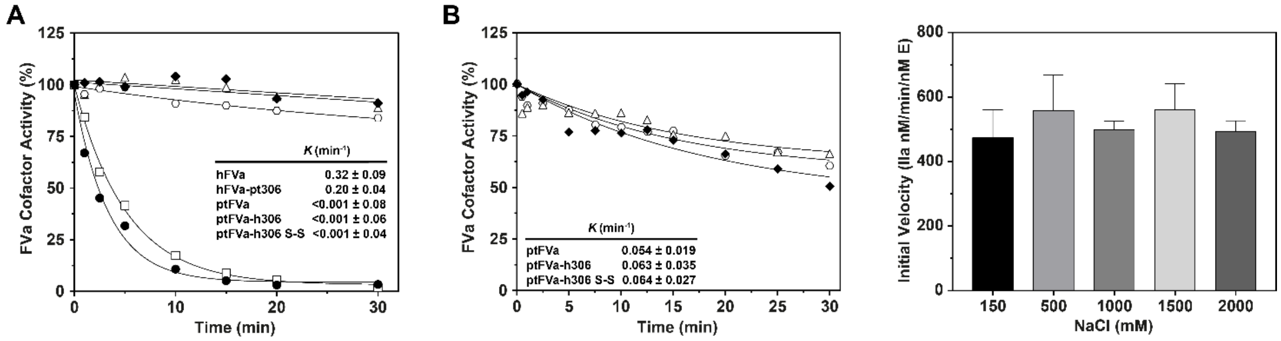

Supplementary Figure IV. Factor Va cofactor activity in conditions of increased temperature or high ionic strength. (A) Reaction mixtures containing 20 nM hFVa (closed circles), hFVa-pt306 (open squares), ptFVa (closed diamonds), ptFVa-h306 (open triangles), or ptFVa-h306 S-S (open hexagons) were incubated at 52°C. At selected time intervals, samples were removed and diluted on ice, upon which they were assayed for cofactor activity employing a FV-specific PT-based clotting assay. **(B)** APC-treated ptFVa (closed diamonds), ptFVa-h306 (open triangles), or ptFVa-h306-SS (open hexagons) were incubated at 52°C. At selected time intervals, samples were removed and diluted on ice, upon which they were assayed for cofactor activity by determining the initial velocity of prothrombin conversion as described in 'Materials and Methods'. Cofactor decay rate constants ± 1 S.D. (insets) were determined by fitting the data to a one-phase decay function by non-linear regression. The data are representative of two to three independent experiments. **(C)** ptFVa-h306 S-S (50 nM) was treated with APC (75 nM) at 37°C in the presence of 50 µM PCPS and increasing concentrations of NaCl (150-2000 mM) in assay buffer. After 5 hours of incubation, samples were removed and diluted in assay buffer for cofactor activity assessment by determining the initial velocity of prothrombin conversion. Data represent the mean ± 1 S.D. and are representative of two independent experiments.

Supplementary Figure V

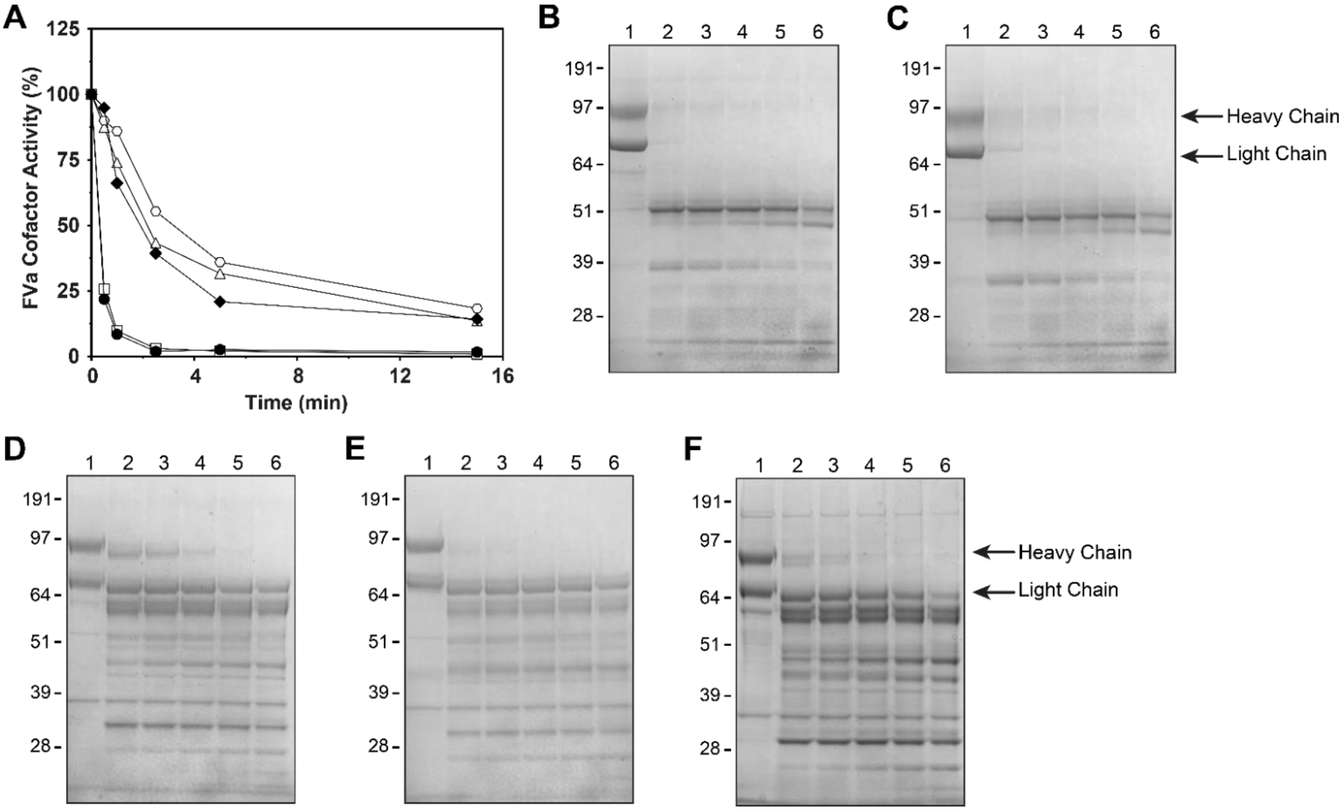

Supplementary Figure V. Plasmin treatment of human and *P. textilis* factor Va variants. (A) Reaction mixtures containing 50 µM PCPS and 500 nM hFVa (closed circles) or hFVa-pt306 (open squares) were incubated with 20 nM plasmin, and similar mixtures containing ptFVa (closed diamonds), ptFVa-h306 (open hexagon), or ptFVa-h306 S-S (open triangles) were incubated with 400 nM plasmin. At selected time intervals, samples were taken and assessed for cofactor activity by determining the initial velocity of prothrombin conversion as detailed in 'Materials and Methods'. The data are representative of two to three independent experiments. SDS-PAGE analysis of plasmin-treated hFVa **(B)**, hFVa-pt306 **(C)**, ptFVa **(D)**, ptFVa-h306 **(E)**, or ptFVa-h306 S-S **(F)** (3 µg per lane) under reducing conditions and visualized by staining with Coomassie Brilliant Blue R-250. Lanes 1-6 represent samples quenched at 0, 0.5, 1, 2.5, 5, and 15 min. The FVa heavy (A1-A2 domains) and light (A3-C1-C2 domains)

chains and the apparent molecular weights of the standards are indicated. The data are representative of two to three independent experiments.

Supplementary Figure VI

Sequence alignment

```
                                    Loop1
Human        AQLRQFYVAAQGISWSYRPEPTNS-SLNLSVTSFKKIVYREYEPYFKKEKPQSTISGLLG   59
P.textilis   AQLREYHIAAQLEDWDYNPQPEELSRLSESDLTFKKIVYREYELDFKQEKPRDALSGLLG   60
             ****::::***  .*.*.*:* :    *. *  :**********  **:***:.::*****

Human        PTLYAEVGDIIKVHFKNKADKPLSIHPQGIRYSKLSEGASYLDHTFPAEKMDDAVAPGRE   119
P.textilis   PTLRGEVGDSLIIYFKNFATQPVSIHPQSAVYNKWSEGSSYSDGTSDVERLDDAVPPGQS   120
             *** .**** : ::*** * :*:*****.  *.* ***:** * *  .*::**** **:.

Human        YTYEWSISEDSGPTHDDPPCLTHIYYSHENLIEDFNSGLIGPLLICKKGTLTEGGTQKTF   179
P.textilis   FKYVWNITAEIGPKKADPPCLTYAYYSHVNMVRDFNSGLIGALLICKEGSLNANGSQKFF   180
             :.* *.*: : **.: ******: **** *::.******** *****:*:*. .*:** *

Human        DKQIVLLFAVFDESKSWSQSSSLMYTVNGYVNGTMPDITVCAHDHISWHLLGMSSGPELF   239
P.textilis   NREYVLMFSVFDESKNWYRKPSLQYTINGFANGTLPDVQACAYDHISWHLIGMSSSPEIF   240
             ::: **:*:******.* :. ** **:**:.***:**:.***:**:******:****.**:*

Human        SIHFNGQVLEQNHHKVSAITLVSATSTTANMTVGPEGKWIISSLTPKHLQAGMQAYIDIK   299
P.textilis   SVHFNGQTLEQNHYKVSTINLVGGASVTADMSVSRTGKWLISSLVAKHLQAGMYGYLNIK   300
             *:*****.*****:***:*.**..:*.**:*:*.   ***:****. ******* .*::**

Human        NCPKKTRNLKKITREQRRHMKRWEYFIAAEEVIWDYAPVIPANMDKKYRSQHLDNFSNQI   359
P.textilis   DCGNPDTLTRKLSFRELMKIKNWEYFIAAEEITWDYAPEIPSSVDRRYKAQYLDNFSNFI   360
             :* :       :*::  .:  ::*.*********: ***** **:.:*::*::*:****** *

Human        GKHYKKVMYTQYEDESFTKHTVNPNMKEDGILGPIIRAQVRDTLKIVFKNMASRPYSIYP   419
P.textilis   GKKYKKAVFRQYEDGNFTKPTYAIWPKERGILGPVIKAKVRDTVTIVFKNLASRPYSIYV   420
             **:***.:: **** .*** *     ** *****:*:*:****:.*****:********

                   Loop2
Human        HGVTFSPYEDEVNSSFTSGRNNTMIRAVQPGETYTYKWNILEFDEPTENDAQCLTRPYYS   479
P.textilis   HGVSVSKDAEGAIYPSDPKENITHGKAVEPGQVYTYKWTVLDTDEPTVKDSECITKLYHS   480
             ***:.*    : .       .* *  :**:**:.*****.:*: **** :*::*:*: *:*

Human        DVDIMRDIASGLIGLLLICKSRSLDRRGIQRAADIEQQAVFAVFDENKSWYLEDNINKFC   539
P.textilis   AVDMTRDIASGLIGPLLVCKHKALSVKGVQNKADVEQHAVFAVFDENKSWYLEDNIKKYC   540
              **: ********* **:** ::*. .*:*. **:**:******************:*:*

Human        ENPDEVKRDDPKFYESNIMSTINGYVPESITTLGFCFDDTVQWHFCSVGTQNEILTIHFT   599
P.textilis   SNPSAVKKDDPKFYKSNVMYTLNGYASDRTEVLRFHQSEVVQWHLTSVGTVDEIVPVHLS   600
             .**. **:******:**:* *:***. :    .* *  .:.****: **** :**: :*::

Human        GHSFIYGKRHEDTLTLFPMRGESVTVTMDNVGTWMLTSMNSSPRSKKLRLKFRDVKCIPD   659
P.textilis   GHTFLSKGKHQDILNLFPMSGESATVTMDNLGTWLLSSWGSCEMSNGMRLRFLDANYDDE   660
             **:*:    :*:* *.**** ***.******:***:*:* .*.  *: :**:* *.:    :

Human        DDEDSYEIFEPPESTVMATRKMHDRLEPEDEESDADYDYQNRLAAALGIR----------   709
P.textilis   DEGNEEEEEDDGDIFADIFIPSEVVKKKEEVPVNFVPDPESDALAKELGLIDDEGNPIIQ    665
             *: :. *  :  :  .                  *:..        *      *.

Human        ----------------------
P.textilis   PRREQTEDDEEQLMKASMLGLR                                          742
```

Supplementary Figure VI. Sequence alignment of the human and *P. textilis* A1-A2 domains in FVa. "*" represents identical amino acids, ":" represents very similar amino acids, "." represents somewhat similar amino acids. The A1, A2, and A3 domains are indicated in blue, pink, and green, respectively. The Arg306 region is

indicated by the black frame and loop1 and loop2 are highlighted in yellow. Black arrows indicate APC cleavage sites.

Supplementary Figure VII

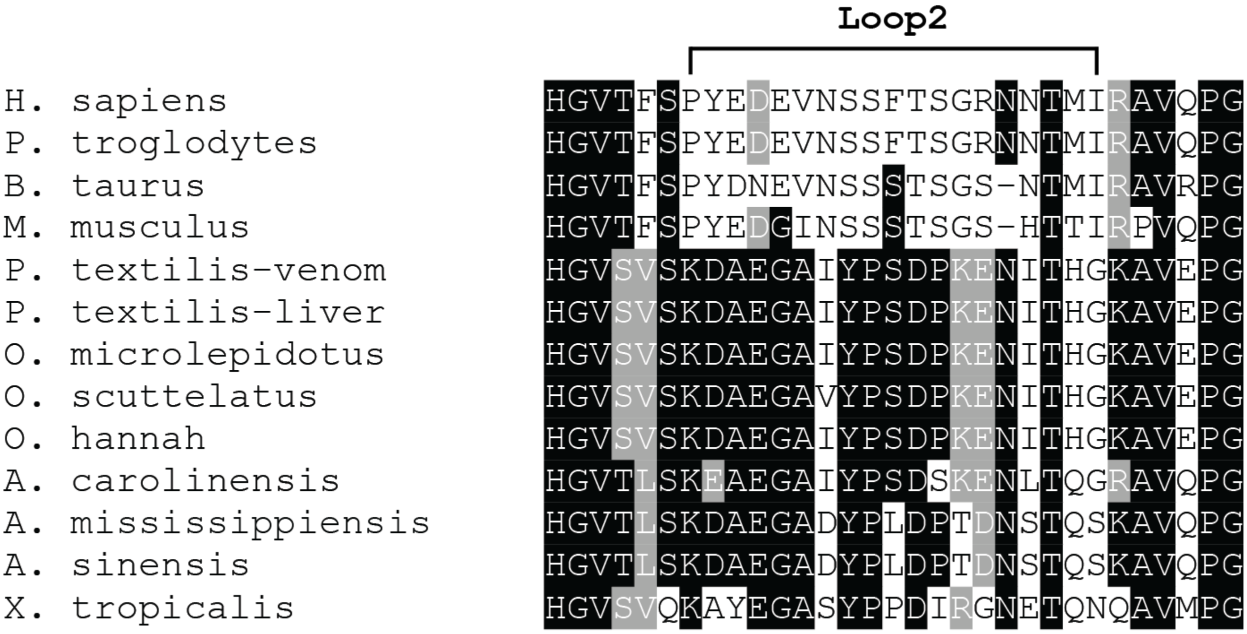

Supplementary Figure VII. Sequence alignment of the A2 domain loop2 in FV. Alignment of the amino acid sequences comprising the FV A2 domain loop2 (Clustal Omega Module; EMBL-EBI, UK). Residues identical to the consensus are shown in black; residues similar to the column consensus are shown in grey. The amino acids corresponding to the A2 domain loop2 are indicated.

Supplementary Figure VIII

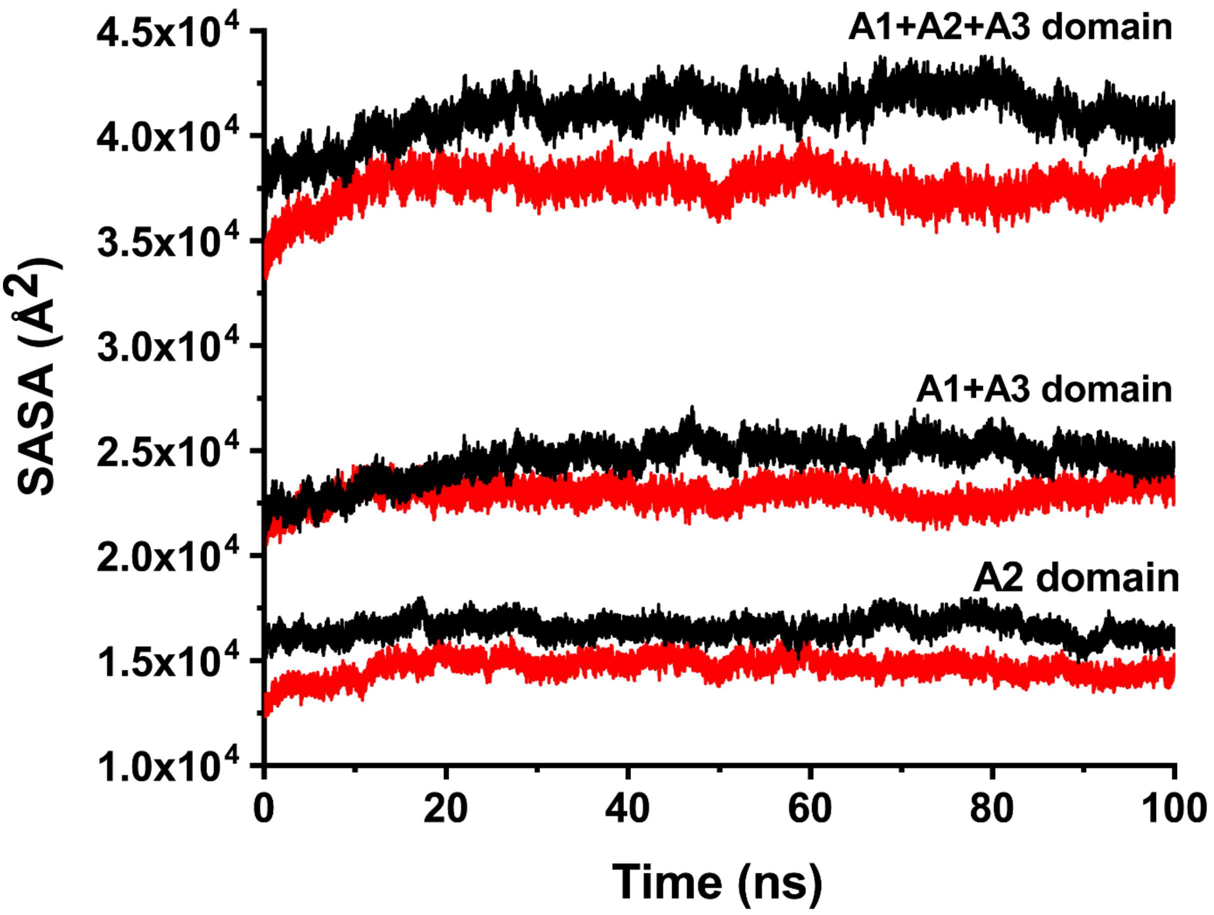

Supplementary Figure VIII. Solvent accessible surface areas (SASA) in human and *P. textilis* FV. The SASA of the A2 domain, the A1 and A3 domain complex, and the A1, A2, and A3 domain complex were calculated as described in 'Materials and Methods'. The black and red lines represent human and *P. textilis* FV, respectively.

Supplementary Table I

	hFV	hFV-pt306	ptFV	ptFV-h306	ptFV-h306 S-S
$K_{d, app}$ (nM)	0.71 ± 0.20	0.97 ± 0.20	0.83 ± 0.18	0.48 ± 0.16	0.97 ± 0.29

Supplementary Table I. Apparent binding affinity for the cofactor. The apparent binding affinity of hFXa or ptFXa for their respective cofactor species was obtained as described in 'Materials and Methods'. The mean values ± S.D. are representative of two to three independent experiments.

Supplementary Table II

Binding surfaces	hFVa	ptFVa-h306 S-S	hFVa-ptloop2
**A2-A1**	Arg439-Glu20*<br>Pro617-Ser240<br>Arg619-Ala257	Lys469-Asn252<br>Glu430-Asn245<br>Tyr419-Asn245<br>Asp464-Thr248<br>Asp462-Lys255<br>Ser620-Asn260<br>Ser424-Arg275	Arg475-Lys254<br>Gly421-Gly245<br>Tyr418-Asn244<br>Asp469-Lys254*<br>Asp480-Asn244<br>Tyr433-Trp278<br>Phe462-Lys254<br>Asp480-Ser281<br>Pro617-Ser240<br>Asn591-Lys286<br>Glu621-His253<br>Arg619-Thr259
**A2-A3**	Ile593-Ser1127<br>Asp611-Gly965<br>Arg648-Glu978*<br>Asp611-Arg1006*<br>Glu610-Arg1017*<br>Gly600-Arg1006<br>Asp611-Lys975*	Gly601-Arg926<br>Asn630-Tyr888<br>Asp629-Arg926*<br>Asp612-Gly885<br>Thr603-Arg926<br>Asn615-Gly1077<br>Asn615-Pro1076<br>Arg649-Asp898*<br>Arg651-Asp898*<br>Gly601-Tyr888<br>Thr633-Lys890<br>Arg651-Asp899*	Ile593-Ser1127<br>Asp611-Arg1006*<br>Asp611-Gly965<br>Glu610-Arg1017*<br>Asn467-Asn1145<br>Arg608-Glu1143*<br>His609-Glu1010<br>Arg648-Glu978*

* Denotes the charged interactions

Supplementary Table II. Key hydrogen bond interaction pairs between the A2 domain and the A1 or A3 domain. Hydrogen bond pairs were identified as described in ‘Materials and Methods’.

Major Resources Table

In order to allow validation and replication of experiments, all essential research materials listed in the Methods should be included in the Major Resources Table below. Authors are encouraged to use public repositories for protocols, data, code, and other materials and provide persistent identifiers and/or links to repositories when available. Authors may add or delete rows as needed.

Animals (in vivo studies)

Not Applicable

Genetically Modified Animals

Not Applicable

Antibodies

Target antigen	Vendor or Source	Catalog #	Working concentration	Persistent ID / URL
Factor V (polyclonal)	Affinity Biologicals	SAFV-IG	1:2000	https://affinitybiologicals.com/pdfs/Factor%20V/SAFV-IG%20-%20Sheep%20Anti-Human%20Factor%20V%20-%20polyclonal%20antibody%20-%20whole%20IgG%20-.pdf
Factor V, Between Arg306 and Arg506	Haematologic Technologies	AHV-5146	1:1000	https://www.haemtech.com/products/antibodies/anti-human-factor-v-antibody-4

DNA/cDNA Clones

Clone Name	Sequence	Source / Repository	Persistent ID / URL
pED-FV810		Vector was a generous gift from Dr. Rodney Camire, University of Pennsylvania	
pED-ptFV		Vector was a generous gift from Dr. Rodney Camire, University of Pennsylvania	

Cultured Cells

Name	Vendor or Source	Sex (F, M, or unknown)	Persistent ID / URL
BHK	unknown	unknown	

Other

Description	Source / Repository	Persistent ID / URL

Q5® Site-Directed Mutagenesis Kit	New England Biolabs	
alpha-Select GOLD Efficiency (DH5)	Bioline	
Miniprep kit	Promega	
Maxiprep kit	Promega	
Lipofectamine 2000	ThermoFisher	
Opti-MEM	ThermoFisher	
DMEM-F12	ThermoFisher	
Fetal Bovine Serum, Qualified	ThermoFisher	
Antibiotic-Antimycotic Solution	ThermoFisher	
Insulin-Transferrin-Sodium Selenite Supplement	ThermoFisher	
G-418 Sulfate	Santa Cruz	
SP-sepharose Fast Flow	Cytiva	
Q-sepharose Fast Flow	Cytiva	
Superdex 200 Increase 10/300 GL	Cytiva	
AKTA Purifier 10	Cytiva	
POROS HQ column	Applied Biosystems	
POROS HQ column	Applied Biosystems	
Start 4 Stollingsanalyzer	Diagnostica Stago	
Factor V-deficient Plasma	Diagnostica Stago	
STA Neoplastin CI plus 10	Diagnostica Stago	
Benzamidine hydrochloride	Sigma	
Coomassie Briliant Blue G250	Sigma	
EDTA >99%	Sigma	
L-α-phosphatidylcholine (Egg, Chicken)	Avanti Polar Lipids	
L-α-phosphatidylserine (Brain, Porcine)	Avanti Polar Lipids	
S-2238	Chromogenix	
RVV-X Activator	Haematologic Technologies	
Human Prothrombin	Haematologic Technologies	
Factor Xa	Haematologic Technologies	
Activated Protein C	Haematologic Technologies	
Protein S	Haematologic Technologies	
Plasmin	Haematologic Technologies	
Thrombin	Haematologic Technologies	
dansylarginine N-(3-ethyl-1,5-pentanediyl)amide	Haematologic Technologies	

Supplemental References

1. Krishnaswamy S, Walker RK. Contribution of the prothrombin fragment 2 domain to the function of factor va in the prothrombinase complex. *Biochemistry*. 1997;36:3319-3330
