# Supplementary figures and images for: ptFVa (Pseudonaja Textilis Venom-Derived Factor Va) Retains Structural Integrity Following Proteolysis by Activated Protein C
Source: Arterioscler Thromb Vasc Biol. 2021 Jun 24;41(8):2263–76. doi: 10.1161/ATVBAHA.121.316038 (PMC8288481; doi:10.1161/ATVBAHA.121.316038)

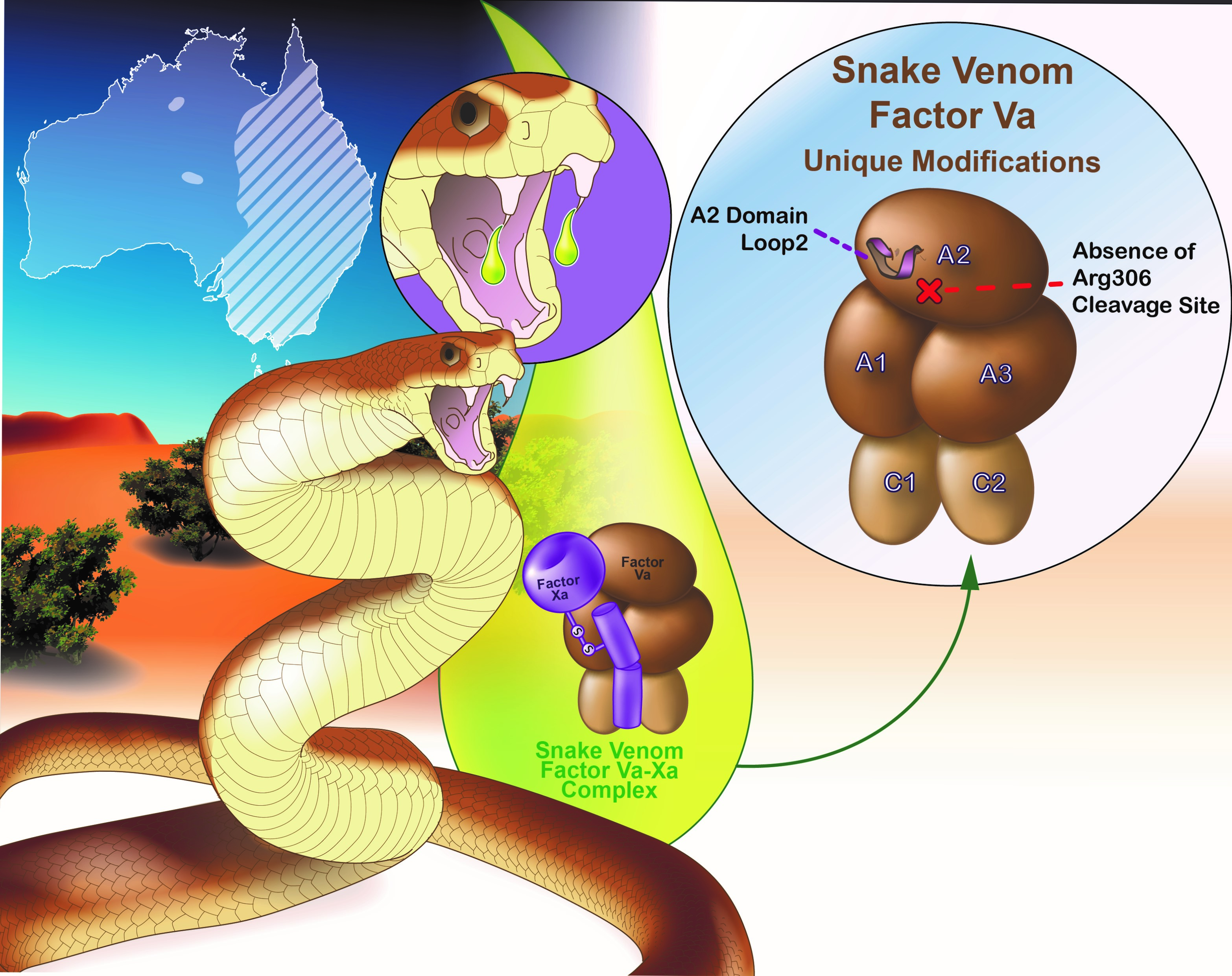

Supplement: Supplementary file 2 [file atv-41-2263-s002.jpg]
